# A comparative study of methods for dynamic survival analysis

**DOI:** 10.3389/fneur.2025.1504535

**Published:** 2025-02-18

**Authors:** Wieske K. de Swart, Marco Loog, Jesse H. Krijthe

**Affiliations:** ^1^Institute for Computing and Information Sciences, Radboud University, Nijmegen, Netherlands; ^2^Pattern Recognition Laboratory, Delft University of Technology, Delft, Netherlands

**Keywords:** survival analysis, dynamic prediction, longitudinal data, landmarking, machine learning, ADNI

## Abstract

**Introduction:**

Dynamic survival analysis has become an effective approach for predicting time-to-event outcomes based on longitudinal data in neurology, cognitive health, and other health-related domains. With advancements in machine learning, several new methods have been introduced, often using a two-stage approach: first extracting features from longitudinal trajectories and then using these to predict survival probabilities.

**Methods:**

This work compares several combinations of longitudinal and survival models, assessing their predictive performance across different training strategies. Using synthetic and real-world cognitive health data from the Alzheimer's Disease Neuroimaging Initiative (ADNI), we explore the strengths and limitations of each model.

**Results:**

Among the considered survival models, the Random Survival Forest consistently delivered strong results across different datasets, longitudinal models, and training strategies. On the ADNI dataset the best performing method was Random Survival Forest with the last visit benchmark and super landmarking with an average tdAUC of 0.96 and brier score of 0.07. Several other methods, including Cox Proportional Hazards and the Recurrent Neural Network, achieve similar scores. While the tested longitudinal models often struggled to outperform simple benchmarks, neural network models showed some improvement in simulated scenarios with sufficiently informative longitudinal trajectories.

**Discussion:**

Our findings underscore the importance of aligning model selection and training strategies with the specific characteristics of the data and the target application, providing valuable insights that can inform future developments in dynamic survival analysis.

## 1 Introduction

Survival analysis has been a cornerstone in medical research for decades, enabling researchers to uncover valuable insights into the prognosis of patients in neurology, cognitive health and health-related domains. Traditionally, the Cox proportional hazards model has been the go-to tool for survival analysis, offering valuable insights into the effects of covariates on time-to-event outcomes.

Recent advancements in machine learning and the collection of more longitudinal data have offered a novel set of approaches for the dynamic prediction of survival outcomes (1–4). With these methods, risk predictions can be updated as new information becomes available, reflecting the evolving nature of patient health data. These machine learning techniques bring the promise of improved predictive accuracy and the potential to unravel complex relationships within cognitive health and other healthcare datasets.

However, there exists a critical need for comprehensive comparison studies that delve into these methods' efficacy and potential shortcomings. One of the inherent challenges in such comparative studies lies in the lack of consistency and transparency in methodology implementation. It is not always clear how various machine learning models are implemented, leading to potential discrepancies in results. Furthermore, the metrics employed for performance assessment often differ between studies, making it challenging to draw meaningful conclusions from these comparisons. Lastly, some machine learning-based survival analysis methods introduce a multitude of new components and hyperparameters, raising questions about which of these components truly drive improvements in predictive performance. Combined, these challenges have led to a lack of clarity on the effectiveness of each introduced modelling component, which complicates the selection of the most suitable model for a given healthcare application.

This paper aims to bridge these gaps by presenting a comprehensive study comparing various machine learning methods for dynamic survival analysis. We seek to provide researchers and practitioners in the field of cognitive health, and healthcare in general, with a clearer understanding of the strengths and weaknesses of these methods and the nuances of their implementation.

By employing a two-stage modeling approach, we can effectively combine different longitudinal and survival models, allowing for a more nuanced comparison of their strengths and weaknesses. In addition, we investigate different training strategies. Using synthetic data with specific characteristics, we aim to assess the relative performance of various models and identify the key factors contributing to their success or limitations.

Our work is motivated by challenges in cognitive health, specifically the prediction of dementia risk. Dementia is an escalating global concern for which risk reduction strategies are crucial, especially given the lack of available curative treatments (5). Several modifiable risk factors have been identified that, if addressed early, could prevent or delay the onset of the disease (6). Improving predictive methods for dementia risk could offer valuable guidance for early interventions and behavioral changes, ultimately helping to reduce the global burden of dementia.

To demonstrate the applicability of the considered methods within neurology, we look at dementia risk prediction using data from the Alzheimer's Disease Neuroimaging Initiative (ADNI).

The main contributions of this study are (i) the comparison of methods for dynamic survival analysis across diverse scenarios, including both statistical approaches, such as MFPCA, and neural network-based methods, (ii) the separate investigation of the contributions to the system performance of longitudinal and survival models by testing multiple combinations and (iii) the comparison of several landmarking strategies that can be used during model training.

This article is structured as follows. In Section 2, we introduce the dynamic prediction problem and provide a description of the relevant methods and datasets. In Section 3, we present the results on synthetic data and data from the ADNI study. In Section 4, we further discuss the results and conclude our work.

## 2 Materials and methods

This section covers the description of the dynamic prediction problem, landmarking techniques, the learning methods used, a description of the synthetic data and the real-world data studied, and the way we evaluate the various approaches. We go through these topics in the order given, but we start with a brief subsection on notation.

### 2.1 Notation

The data consists of a set of *N* subjects with *Q* covariates measured at multiple time points. Each subject *i* has a set of covariates *Y*_*iq*_(*t*_*ij*_) measured at each visit *j*, at measurement time *t*_*ij*_, for a total of *J*_*i*_ visits. Each subject is observed until either the time of the event of interest ($T_{i}^{*}$) or the time at which the subject is censored (*C*_*i*_). The observed event time is $T_{i}=min\left(T_{i}^{*},C_{i}\right)$ and the event indicator δ_*i*_ identifies whether the event occurred (δ_*i*_ = 1) or the subject was censored (δ_*i*_ = 0).

Our goal is to make a prediction of the probability of survival from so-called landmark times *l* to future prediction times *t*>*l*, conditional on survival at landmark time *l*, based on the data up until time *l*. We will use $Y_{i}^{\left(l\right)}$ to indicate the subset of the measurements of subject *i* up until time *l*. The conditional survival function is then given by:


(1) $$\begin{array}{l} S\left(t|Y_{i}^{\left(l\right)}\right)=P\left(T_{i}^{*}>t|T_{i}^{*}>l,Y_{i}^{\left(l\right)}\right). \end{array}$$

Some models instead estimate the hazard function λ(*t*), which is the instantaneous risk that the event will occur. The hazard function is related to the survival function, so that


(2) $$\begin{array}{l} S\left(t\right)=\exp\left[-\int_{0}^{t}\lambda \left(u\right)du\right]. \end{array}$$

### 2.2 Dynamic prediction

Rather than a static prediction at a single landmark time, we want dynamically updated predictions at the next landmark times, as more covariate measurements become available for the subject. Two popular strategies in dynamic survival analysis are landmarking and joint modeling (7, 8).

Landmarking (9) offers a straightforward approach by building separate survival models at pre-defined landmark times. For each landmark *l* a survival model is fit using the subjects that are still at risk at that time. In the traditional landmarking approach only the last available measurement of each subject is included (7). This method is easy to implement and execute, since it only involves fitting normal survival models, and avoids making assumptions about the joint distribution of longitudinal and survival data. However, it ignores information that could be available in the history of the covariates.

Joint modeling integrates the longitudinal and survival processes by simultaneously modeling the time-dependent covariates and the event times using shared random effects (10, 11). This method accounts for the direct relationship between longitudinal measurements and survival, often leading to more accurate risk predictions if the models are correctly specified. Unfortunately, including multiple longitudinal covariates in a joint model is computationally difficult, because of the increasing dimensionality in the random effects (12).

To address the limitations of both strategies, a two-stage approach has emerged that combines the flexibility of landmarking and the comprehensive nature of joint modeling (13, 14). In the first stage, a longitudinal model is used to model the trajectories of the time-varying covariates. In the second stage, the predictions of the longitudinal model are incorporated into a survival model. With this approach, dynamic risk estimates can be obtained without the computational complexity associated with joint modeling. This strategy can be found in many recent works on dynamic survival analysis (1, 3, 15, 16) and will also be used here. An additional advantage of this two-stage approach is that it allows for the independent evaluation of the benefits of specific longitudinal and survival methods. The longitudinal and survival models examined in this study will be detailed in Sections 2.4, 2.5, respectively.

To evaluate a model's accuracy in dynamically predicting survival probabilities, performance is typically assessed at multiple landmark times. This approach reflects real-world scenarios, where patients visit a doctor at different points in their life. For each landmark time, predictions are made for future time points using only the subjects who are still at risk at that landmark time and who were not used to train the model. Only the longitudinal data available up to the landmark time is used for these predictions.

However, in the context of this two-stage approach, the best way to handle these landmark times during training remains unclear. Some studies fit a single two-stage model for all landmark times, training it on the complete longitudinal trajectories (15, 17). In contrast, Gomon et al. (16) argued that the more appropriate method is to fit a separate model for each landmark, similar to the traditional landmarking approach. In this work, we further explore the differences between these strategies and introduce alternative methods for handling landmarking during training.

### 2.3 Landmarking methods

This section describes the two landmarking methods formalized by Gomon et al. (16) and expands on their work by introducing two additional approaches. Following Gomon et al. (16), we refer to the process of selecting subjects that are still at risk at time *l* (*T*_*i*_>*l*) and truncating their data to obtain $Y_{i}^{\left(l\right)}$ as *landmarking* the data at time *l*.

#### 2.3.1 No landmarking

With this approach, a single model is trained for all landmark times, using the entire longitudinal history $Y_{i}^{\left(J_{i}\right)}$ for each subject. By incorporating all available data, the model has the most information to learn from. However, as Gomon et al. (16) demonstrated, this strategy, which they refer to as relaxed landmarking, can introduce bias due to discrepancies between the training and evaluation data. Specifically, at later landmark times, the training data includes subjects who have already experienced the event. This could create a mismatch between the training and evaluation population, since during evaluation only subjects still at risk are included.

Another concern is that models trained on entire longitudinal trajectories can use information that is not available during evaluation. Observational data typically includes measurements until shortly before an event or censoring occurs. Without landmarking, the training data may therefore contain information up to the event time *T* and there is nothing that prevents a model during training to mainly use the latest available information for prediction. As a result, it is unclear what happens during evaluation if these measurements close to *T* are unavailable. There is at least little reason to believe that the trained model has also learned how to deal with this limited-information scenario.

#### 2.3.2 Strict landmarking

With the strict landmarking approach a separate model is trained for each landmark time *l*, where the data is landmarked at time *l* (16). In this case, the training data is processed the same way as the evaluation data, reducing the risk of bias caused by differences between the two sets.

However, landmarking the training set removes a substantial amount of data, making it harder to learn robust patterns. At early landmark points, truncating the training data could discard relevant patterns that emerge later in these longitudinal trajectories. At later landmark times, excluding subjects who have already experienced the event can greatly reduce the number of training samples, increasing model variance. Although these subjects are no longer at risk, their early trajectories may still hold valuable insights for prediction. In observational datasets, subjects typically enter the study at different stages of disease progression. This means that subjects may not be directly comparable at baseline or at any chosen landmark time. Therefore, patterns from one subject's earlier trajectory may provide useful insights into a later stage of another subject's trajectory. Consequently, this strict landmarking approach risks discarding important data that could improve prediction accuracy during evaluation.

#### 2.3.3 Super landmarking

In Van Houwelingen and Putter (18), the use of a super landmarking dataset is proposed as an extension of the traditional landmarking strategy. With this method, a separate landmarked dataset is created for each chosen landmark time and the resulting subsets are combined into a single dataset. As a result, subjects are repeatedly included in the dataset, as long as they remain at risk at each landmark time, with an increasing number of longitudinal measurements at later landmarks. This approach eliminates the need to train a separate model for each landmark time.

Although this method was introduced for traditional landmarking some time ago, we have not yet seen any applications for two-stage methods. Nevertheless, it could strike a good balance between retaining enough information to learn relevant patterns and avoiding the overfitting issues that arise when no landmarking method is used.

A potential drawback of this approach is the increased size of the training dataset, especially when many landmark points are used. This could result in longer training times, particularly since some models take over twice as long to train when the dataset doubles in size.

#### 2.3.4 Random landmarking

In many real-world scenarios a risk prediction for a patient could be desired at any of their visits. If we wish to create a model that performs well for each possible visit time it might be necessary to choose a large set of landmark times. For strict landmarking this would lead to a large set of models, some of which will be trained on a small amount of data. For super landmarking this would result in a large training set with many similar samples. The iterative training process of neural networks offers another option for landmarking, where the training set is transformed differently for each iteration. This novel design is inspired by the use of on-the-fly data augmentation (19), where a random transformation is applied to the data during each training iteration. In this case, in each iteration and for each subject *i* we sample a random number *l* between 1 and *J*_*i*_. We then temporarily truncate the longitudinal data for this subject to $Y_{i}^{\left(l\right)}$, thereby allowing the model to learn from variable input lengths and time frames. This iterative approach ensures that the model is not overfit to any specific time point or landmark, resulting in a more flexible model capable of generalizing across different stages of longitudinal data.

The advantages of the random landmarking method become particularly evident when predictions are required for a large set of landmark points. By training a single model on a standard-sized training set this method avoids the computational burden of handling either an excessively large dataset or multiple models. This not only reduces training time but also simplifies the implementation process. Additionally, the random selection of visit times in the training set enables the model to generalize across the entire data distribution, rather than specializing in specific landmark points. This combination of computational efficiency and robust predictive performance makes this method especially valuable in large-scale or resource-constrained settings. However, this approach does come with potential trade-offs. First, the inclusion of all subjects, irrespective of their event time, may still create a mismatch between the population used for training and the population used for evaluation. Moreover, while this general model can be more flexible, when evaluated at specific landmark times it could be less effective than a model trained for that specific landmark time.

### 2.4 Longitudinal models

As already mentioned in Section 2.2, we use a two-stage approach for dynamically predicting survival probabilities. Here we will describe the first stage, where one of four longitudinal models will be used to summarize subject trajectories into an encoding *Z*. In the second stage, this encoding is used to train a survival model. These survival models will be described in the next section.

We include a varied set of methods that represent different approaches to encoding longitudinal data. We include two benchmark methods: baseline and last visit. In addition, we include Multivariate Functional Principal Component Analysis (MFPCA), a dimensionality reduction technique which has recently become popular for dynamic survival analysis (4, 15–17). Lastly, we include a Recurrent Neural Network (RNN), as a neural network approach that is suitable for smaller datasets with sequential data (Mienye et al., 2024). The RNN has previously been used for dynamic survival analysis in Lee et al. (1).

#### 2.4.1 Benchmarks

To explore the added value of incorporating longitudinal data, we introduce two benchmark models. The first is termed **Baseline**, which selects the measurements taken at the first time point. This benchmark serves as a reference for traditional survival analysis without the integration of longitudinal data. For the baseline model the encoding for subject *i* is given by


(3) $$\begin{array}{l} Z_{i}=Y_{i}\left(t_{i0}\right). \end{array}$$

The second benchmark, labeled **Last Visit**, selects the measurements taken at the last available time point at or before the landmark time. This approach aligns with the traditional landmarking strategy. For this benchmark the encoding of subject *i* is given by


(4) $$\begin{array}{l} Z_{i}=Y_{i}\left(\max\left\{t_{ij}|t_{ij}<=l\right\}\right). \end{array}$$

#### 2.4.2 MFPCA

Multivariate Functional Principal Component Analysis (MFPCA) is a method that captures the temporal dynamics of longitudinal data by representing them in a lower-dimensional space using functional principal component analysis techniques (20). Similar to traditional principal component analysis, MFPCA decomposes longitudinal trajectories into principal components, allowing for dimensionality reduction while preserving the key features of the data. Several earlier works have used this method to extract features from longitudinal data that can be used in a survival model (4, 16, 17).

For a single longitudinal variable *q* the trajectory *Y*_*iq*_(*t*) can be approximated using the following decomposition


(5) $$\begin{array}{l} Y_{iq}\left(t\right)=\mu_{q}\left(t\right)+\overset{M_{q}}{\underset{m=1}{\sum }}\xi_{iqm}\phi_{qm}\left(t\right), \end{array}$$

where μ_*q*_(*t*) is the mean function, ξ_*iqm*_ are the FPC scores, with nonincreasing variance λ_*qm*_, and ϕ_*qm*_ are the eigenfunctions. The scores ξ_*qm*_ have traditionally been estimated by numerical integration, but this is often not a good approximation if there are only a few measurements available for each subject. Therefore the Principal Analysis by Conditional Estimation algorithm is used, which, under Gaussian assumptions, obtains a better estimation of the FPC scores ξ_*qm*_ (21).

For multiple longitudinal variables, a two-step approach is used, as described in Happ and Greven (22). In the first step, univariate FPCA is estimated for each variable. Every instance is then represented by all its univariate FPC scores. Subsequently, in a second step, the multivariate FPC scores are obtained through a principal component decomposition of these initial univariate FPC scores. With this process, we can transform the *Q* sets of *M*_*q*_ univariate scores of a subject to one set of *M* multivariate scores ρ_*im*_.

We fit the model using the training set to obtain estimates of ${\hat{\lambda }}_{qm}$, ${\hat{\phi }}_{qm}$, ${\hat{\Sigma }}_{Y_{iq}}^{-1}$ and ${\hat{\mu }}_{q}$. The scores ξ_*iqm*_ can then be obtained for any longitudinal covariate *Y*_*iq*_ in the training set or evaluation set using.


(6) $$\begin{array}{l} \xi_{iqm}=E\left[\xi_{iqm}|Y_{iq}\right]={\hat{\lambda }}_{qm}{\hat{\phi }}_{iqm}{\hat{\Sigma }}_{Y_{iq}}^{-1}\left({\text{Y}}_{iq}-{\hat{\mu }}_{q}\right). \end{array}$$

These can then be transformed to obtain the multivariate scores ρ_*im*_. Combined with the baseline variables these scores form the encoding that will be used for the survival prediction. For subject *i* this results in


(7) $$\begin{array}{l} Z_{i}=\left[Y_{\text{i},\text{baseline}}\left(t_{i0}\right),\rho_{i}\right]. \end{array}$$

#### 2.4.3 RNN

Longitudinal data can be represented as sequences, with the set of measurements at each time point corresponding to a step in the sequence. Recurrent Neural Networks (RNNs) are a class of neural networks well-suited for sequential data analysis, making them a natural choice for modeling longitudinal data. RNNs maintain a hidden state that evolves over time, allowing them to capture temporal dependencies in the data.

In this work, we opted to utilize a traditional RNN, specifically a multi-layer Elman RNN (23), instead of more complex architectures like Long Short-Term Memory (LSTM) networks (24). This decision was driven by the desire to focus on more elementary versions of the models, allowing for a clearer comparison of their performance and capabilities.

For each visit *j*, each layer *n* of the RNN computes the following function


(8) $$h_{nj}=\tanh(h_{n-1,j})\;\cdot \;W_{n0}^{T}+b_{n0}+h_{n,j-1}\;\cdot \;W_{n1}^{T}+b_{n1})$$

For the first layer *h*_*n*−1, *j*_ = *Y*(*t*_*j*_), the covariate measurements of a subject at time point *j*, and for each subsequent layer it is the output of the previous layer. *W*_*n*_ and *b*_*n*_ are the weights and bias of the *n*-th layer and *h*_*n, j*−1_ is the hidden state at the previous time point. The final hidden states are combined with the baseline variables and used to make both a longitudinal and a survival prediction. For the following we use *h*_*ij*_ to indicate the final hidden state of subject *i* at time *t*_*ij*_.

For the longitudinal prediction, a feed-forward neural network is used to predict the input variables of the next time point Ŷ_*i*_(*t*_*i, j*+1_) from the hidden state *h*_*ij*_:


(9) $$\begin{array}{l} {Ŷ}_{i}\left(t_{i,j+1}\right)=\text{FNN}\left(h_{ij},X_{i,\text{baseline}}\left(t_{i0}\right)\right) \end{array}$$

For the survival prediction, we extract the hidden state *h*_*i*_*J*__*i*__ at the last time point *J*_*i*_ for each subject *i*. This is combined with the baseline variables to obtain the following encoding for subject *i*


(10) $$\begin{array}{l} Z_{i}=\left[X_{i,\text{baseline}}\left(t_{i0}\right),h_{iJ_{i}}\right]. \end{array}$$

To train the RNN model we use a combined loss function *L* = *L*_long_+*L*_surv_. The longitudinal loss *L*_long_ is the mean squared error between the predicted input variables Ŷ_*i*_(*t*_*ij*_) and the data *Y*_*i*_(*t*_*i, j*+1_):


(11) $$\begin{array}{l} L_{\text{long}}=\frac{1}{N\cdot Q}\overset{N}{\underset{i=1}{\sum }}\overset{Q}{\underset{q=1}{\sum }}\frac{1}{J_{i}}\overset{J_{i}}{\underset{j=2}{\sum }}{\left({Ŷ}_{iq}\left(t_{ij}\right)-Y_{iq}\left(t_{ij}\right)\right)}^{2} \end{array}$$

The survival loss *L*_surv_ is the negative log-likelihood of the time-to-event predictions, which we will formalize in Section 2.5.3.

Unlike the MFPCA model that does not incorporate survival information, the combined loss function used to train the RNN allows the model to prioritize features from the longitudinal trajectories that are more relevant for survival prediction. To investigate its benefit further, we also train a version of the RNN model using only the longitudinal loss, which will be called RNN_long.

### 2.5 Survival models

With each of the longitudinal methods we can transform the longitudinal covariates *Y*_*i*_(*t*_*ij*_) of a subject into an encoding *Z*_*i*_. Subsequently, a survival method is used to predict the conditional survival function from this encoding. For this second-stage model, we consider Cox proportional hazard model, Random Survival Forest (RSF) and a neural network, as these are the most common survival models used in the literature on two-stage methods (4, 15, 16, 25).

#### 2.5.1 Cox Proportional Hazards model

The Cox Proportional Hazards (CPH) model (26) is a classical survival analysis method that estimates the hazard function as a function of covariates while assuming proportional hazards over time. It has been widely used in medical research due to its interpretability and simplicity. In the Cox Proportional hazards model the hazard for subject *i* is defined as


(12) $$\begin{array}{l} \lambda_{i}\left(t\right)=\lambda_{0}\left(t\right)\exp\left(Z_{i}\cdot \beta \right). \end{array}$$

Here *Z*_*i*_ is the encoding of subject *i* given by the longitudinal model.

During training, estimates for β are obtained by minimizing the negative partial log-likelihood on the training data (27). During evaluation, a prediction of the survival function for subject *i* is obtained using


(13) $$\begin{array}{l} S\left(t|Z_{i}\right)=S_{0}{\left(t\right)}^{\exp\left(Z_{i}\cdot \beta \right)}, \end{array}$$

where *S*_0_(*t*) is the estimate of the baseline survival function, obtained using Breslow's estimator (28).

#### 2.5.2 Random Survival Forest

Random Survival Forest (RSF) (29) is an ensemble learning method that extends the traditional random forest algorithm to survival analysis tasks. Instead of decision trees the RSF uses survival trees, which maximize the survival difference between groups. RSF constructs a large number of survival trees on bootstrapped samples of the dataset.

During training, each tree is grown using a bootstrap sample drawn from the training data. At each node of the tree the samples are divided in two groups using the log-rank splitting rule. For this split a random subset of the variables is searched to find the value of one of these variables that maximizes the survival differences between the groups.

During evaluation a prediction of the survival function for subject *i* is obtained for each tree by determining the leaf node for the encoding *Z*_*i*_ and then estimating the survival function of the bootstrap samples in this leaf node using the Kaplan-Meier estimator. The ensemble survival function is obtained by averaging the predictions of all trees.

By aggregating predictions from individual trees, RSF provides robust estimates of survival probabilities and handles complex, nonlinear relationships between features and survival outcomes.

#### 2.5.3 Neural network

For the RNN longitudinal models we also use a feed-forward neural network that is trained with a negative log-likelihood loss function. This network takes the encoding from the longitudinal model as input and, after a softmax layer, outputs a vector of probabilities $\hat{P}\left(T=t|Z_{i}\right)$ for each subject. This indicates the probability of subject *i* having the event of interest during the time interval [*t, t*+1).

From these probabilities, we can compute the failure function at time τ as follows


(14) $$\begin{array}{l} \hat{F}\left(\tau \right)=\frac{\sum_{l<t\leq \tau }\hat{P}\left(T=t\right)}{1-\sum_{t=0}^{l}\hat{P}\left(T=t\right)}=\underset{l<t\leq \tau }{\sum }\frac{\hat{P}\left(T=t\right)}{\hat{P}\left(T>l\right)}\\ \;=\underset{l<t\leq \tau }{\sum }\hat{P}\left(T=t|T>l\right) \end{array}$$

and the survival function is then given by $Ŝ\left(t\right)=1-\hat{F}\left(t\right)$.

These predictions are subsequently used to calculate the following negative log-likelihood loss function


(15) $$\begin{array}{l} L_{NLL}=-\overset{N}{\underset{i=1}{\sum }}\delta_{i}\cdot \log\left(\hat{P}\left(T=T_{i}|Z_{i}\right)\right)+\left(1-\delta_{i}\right)\cdot \log\left(Ŝ\left(T_{i}|Z_{i},T>l\right)\right), \end{array}$$

where *T*_*i*_ indicates the time interval in which an event is observed for subject *i*. In Zadeh and Schmid (30), it is shown that this loss function results in well-calibrated optimization of the survival probabilities.

### 2.6 Simulation study

We developed several simulation scenarios for observational data, inspired by the simulation study described in Lin et al. (15). Through these specific scenarios, we investigate the impact of model misspecification on survival models and examine how effectively longitudinal models can accommodate variations in longitudinal trajectories.

For each scenario, we use *N* subjects with *K* baseline covariates *B*_*ik*_ and *Q* longitudinal covariates *Y*_*iq*_(*t*_*ij*_) measured over a maximum of *J*_*i*_ = 21 visits equally spread between times [0, 10]. The measured longitudinal covariates are given by the true underlying value *X*_*iq*_(*t*_*ij*_) and measurement error as follows


(16) $$\begin{array}{l} Y_{iq}\left(t_{ij}\right)=X_{iq}\left(t_{ij}\right)+\epsilon_{ijq}, \end{array}$$

where ϵ_*ijq*_ was sampled from a standard normal distribution *N*(0, 1). In the subsections below, we provide a further specification of the different scenarios, where we make our choice of covariates and hazard functions precise.

Once we define the hazard the survival function can be obtained using


(17) $$\begin{array}{l} S_{i}\left(t\right)=\exp\left[-\int_{0}^{t}\lambda_{i}\left(u\right)du\right]. \end{array}$$

The survival time for every subject is determined by generating *u* from the standard uniform distribution and determining the first visit time *t* at which *S*_*i*_(*t*) < *u*. In addition, we independently simulate censoring times from the uniform distribution *U*(1, 22). The observed event time is then the minimum of the survival and censoring times.

#### 2.6.1 Scenario 1

In this baseline scenario, based on Lin et al. (15), we consider covariates that evolve linearly over time, and proportional hazards. For the longitudinal covariates the following submodel is used:


$$X_{iq}\left(t_{ij}\right)=\beta_{0q}+\beta_{1q}x_{iq}+\beta_{2q}t_{ij}+b_{iq}$$

We create 3 covariates with coefficients β_0_ = [1.5, 2, 0.5], β_1_ = [2, −1, 1] and β_2_ = [1.5, −1, 0.6], scalar covariate *x*_*iq*_~*N*(3, 1) and subject-specific random effects *b*_*iq*_~*N*(0, Σ), where


$$\Sigma =\left[\begin{array}{ccc} \sigma_{1}^{2} & \eta_{12}\sigma_{1}\sigma_{2} & \eta_{13}\sigma_{1}\sigma_{3}\\ \; & \sigma_{2}^{2} & \eta_{23}\sigma_{2}\sigma_{3}\\ \; & \; & \sigma_{3}^{2} \end{array}\right],$$

with σ = [1, 1.5, 2] and [η_12_, η_13_, η_23_] = [−0.2, 0.1, −0.3].

The hazard function for this scenario is given by


$$\lambda_{i}\left(t\right)=\lambda_{0}\left(t\right)\exp\left[\gamma B_{i}+\overset{3}{\underset{q=1}{\sum }}\alpha_{q}X_{iq}\left(t_{ij}\right)\right].$$

Where the baseline hazard λ_0_(*t*) = exp(−7). We use two baseline covariates, with γ = [−4, 2] and *B*_*i*_ = [*z*_*i*1_, *z*_*i*2_], with *z*_1_~*Bin*(*p* = 0.5) and *z*_2_~*N*(0, 1). For the longitudinal trajectories we set α = [0.2, −0.2, 0.4].

#### 2.6.2 Scenario 2

Similar to Lin et al. (15), in the second scenario, an interaction term is added to the baseline covariates to investigate the effect of model misspecification. In this case the baseline covariates are defined by *B*_*i*_ = [*z*_*i*1_, *z*_*i*2_, *z*_*i*1_·*z*_*i*2_] with γ = [−4, 2, 4], where the interaction term is not used as a predictor in the models.

#### 2.6.3 Scenario 3

Although the scenarios given above incorporate several longitudinal covariates, these covariates follow similar trajectories for all subjects. In fact, for these scenarios it is possible to model the time-varying component within the baseline hazard function. It would therefore be possible to learn the model of scenario 1 using a Cox Proportional Hazard model fitted with the values of the covariates at baseline [*B*_*i*_ and *X*_*i*_(0)]. The derivation for this can be found in the Supplementary material.

Since the measurements of the observed longitudinal covariates *Y*_*iq*_(*t*_*ij*_) contain independently sampled measurement error ϵ_*ijq*_, having access to multiple of these measurement could improve the estimate of the underlying covariates *X*_*iq*_(*t*_*ij*_). Still, longitudinal models that are capable of extracting information about how covariates change over time do not have a large advantage in these first scenarios.

To investigate how well the different methods can model differences over time we make a modification to the submodel for the longitudinal covariates by combining the subject specific random effects in the time-dependent component. For each subject the covariates will still have a constant slope, but this slope now does differ per subject:


$$X_{iq}\left(t_{ij}\right)=\beta_{0q}+\beta_{1q}x_{iq}+\left(\beta_{tq}+b_{iq}\right)t_{ij}.$$

#### 2.6.4 Scenario 4

In the previous scenarios all longitudinal covariates have a direct, immediate effect on the hazard. Additionally, the values of these covariates change linearly over time, making it relatively easy to predict future values. In our last scenario we increase the complexity of the data generating process by adding a longitudinal covariate with random values, which has a delayed effect on the hazard. The random nature of this covariate makes it impossible to accurately predict future values. By delaying the effect on the hazard it is still possible to determine future values of the hazard based on historical values of the covariates. However, knowledge of the survival prediction might be required to transform the longitudinal covariate in the correct way.

The additional longitudinal covariate is sampled from the uniform distribution, given by


$$X_{i4}\left(t_{ij}\right)\sim U\left(-11,9\right).$$

Afterwards we apply the following transformation, which takes the sum of this covariate and delays this by 6 visits (corresponding to 3 years)


$$R_{i}(t_{ij})=\{\begin{array}{ll} 0 & \text{if }j<=6\\ \sum_{k=0}^{j-7}X_{i4}(t_{ik}) & \text{if }j>6 \end{array}$$

The hazard function for this scenario is then given by


$$\lambda_{i}\left(t\right)=\lambda_{0}\left(t\right)\exp\left[\gamma B_{i}+\overset{3}{\underset{q=1}{\sum }}\alpha_{q}X_{iq}\left(t_{ij}\right)+0.2\cdot R_{i}\left(t_{ij}\right)\right].$$

With this scenario we investigate whether a model can detect the effect of a covariate, even if the covariate itself cannot be accurately modelled.

### 2.7 Application: ADNI

To demonstrate performance in a real-world data analysis setting for cognitive health, we use data from the Alzheimer's Disease Neuroimaging Initiative (ADNI). This is an ongoing longitudinal observational study that was launched in 2003. The primary goal of ADNI has been to test whether serial magnetic resonance imaging (MRI), positron emission tomography (PET), other biological markers, and clinical and neuropsychological assessment can be combined to measure the progression of mild cognitive impairment (MCI) and early Alzheimer's disease (AD). More information about ADNI is available on the website: https://adni.loni.usc.edu/.

Our goal is to predict the time to a dementia diagnosis. Subjects have regular follow-up visits and at each of these a diagnosis is given. The event time will either be the time of the first visit where a dementia diagnosis is given or the time of the last available visit where a non-dementia diagnosis is given. Subjects that already have a dementia diagnosis at baseline are removed from the dataset. Several other works have used the ADNI study to evaluate dynamic survival predictions using a similar preprocesssing strategy for the data (2, 15–17).

This work uses the TADPOLE data set^1^ constructed by the EuroPOND consortium (31). This is a fixed version of the ADNI dataset, created for a classification challenge, and therefore allows for easier reproduction of the results. After exclusion of subjects with dementia at baseline this dataset consists of 1,390 subjects. We included 7 demographic baseline features and 15 longitudinal variables, which are described in Table 1.

**Table 1:** Description of the included features from the ADNI TADPOLE dataset.

	**Name**	**Description**
Baseline	APOE4	Number of apolipoprotein ϵ4 alleles
Baseline	PTEDUCAT	Years of education
Baseline	PTETHCAT	Ethnicity
Baseline	PTGENDER	Gender
Baseline	PTMARRY	Marriage status
Baseline	PTRACCAT	Race
Longitudinal	AGE_t	Subject age at visit time
Longitudinal	ADAS11	Alzheimer's disease assessment scale (11-item)
Longitudinal	ASAS13	Alzheimer's disease assessment scale (13-item)
Longitudinal	CDRSB	Clinical dementia rating (sum of boxes)
Longitudinal	Entorhinal	MRI measure of entorhinal
Longitudinal	Fusiform	MRI measure of fusiform
Longitudinal	Hippocampus	MRI measure of hippocampus
Longitudinal	ICV	MRI measure of ICV
Longitudinal	MMSE	Mini-mental state examination
Longitudinal	MidTemp	MRI measure of MidTemp
Longitudinal	RAVLT_forgetting	Rey auditory verbal learning test (forgetting)
Longitudinal	RAVLT_immediate	Rey auditory verbal learning test (immediate)
Longitudinal	RAVLT_learning	Rey auditory verbal learning test (learning)
Longitudinal	RAVLT_perc_forgetting	Rey auditory verbal learning test (forgetting)
Longitudinal	Ventricles	MRI measure of ventricles
Longitudinal	WholeBrain	MRI measure of WholeBrain

Study participants were followed in 6 month intervals, up to a maximum of 10 years. For most participants visits did not take place every 6 months, with a median number of visits of 7 and a median follow-up time of 3 years. In addition, not all variables were recorded every visit. We impute these missing variables using the last recorded measurement of that subject, following the method of last observation carried forward.

### 2.8 Hyperparameters

Machine learning models often include hyperparameters, which are configuration settings defined before the training process begins. Unlike model parameters, which are learned from the data during training, hyperparameters control aspects such as model architecture, regularization, learning rates, and optimization techniques. These settings can influence the performance of the model, affecting its ability to generalize to new data.

For this study, we opted to use a fixed set of hyperparameters across all models to maintain experimental feasibility and allow for fair comparison. All methods are implemented in Python and the implementation is available at https://github.com/Wieske/DSA_comparison. The hyperparameters were selected based on default values in the model implementations, previous literature, and insights from earlier experiments. Below, we summarize the chosen settings for each model.

For MFPCA the only hyperparameter choice is the number of multivariate scores that will be used. For this we chose to retain the minimum number of multivariate scores that explains at least 95% of the variance in the longitudinal variables.

For the RSF model we set the number of estimators to 1,000 (29), as increasing this value generally improves performance until a plateau is reached, at the cost of longer training time. Additionally, we set the minimum number of samples required to split a node to 32 and the minimum number of samples per leaf node to 16. These settings smooth the model's predictions and prevent overfitting by avoiding overly small nodes.

For neural networks, several hyperparameters were required to define both the architecture and training process. For the architecture of the RNN we set the number of layers to 2, each with a number of nodes equal to 5+ the number of longitudinal covariates. A dropout rate of 0.3 was applied to mitigate overfitting (32). The neural network for the survival prediction contains 2 fully connected layers with 32 hidden nodes. A ReLU activation function and dropout is applied after the first layer, while a softmax function is used after the second layer to obtain probability outputs. To train the neural networks we used an Adam optimizer (33) with a learning rate of 1*e*−3 and weight decay of 1*e*−5, which generally improves generalization performance (34). Each model is trained in batches of size 32 for 100 epochs.

To test the sensitivity of the models to the chosen set of hyperparameters we include an experiment with random hyperparameter optimization (35) on the ADNI dataset in a limited setting. For each parameter we set a range or distribution, which includes the chosen value. For instance, the number of layers in a neural network is now chosen randomly from 1, 2, and 3. We then generate 100 sets of hyperparameters, where for each parameter a value is randomly sampled from the chosen range or distribution. We train each model with this parameter set on 80% of the training set, using the other 20% to validate the results. The model with the best results on this validation set is then evaluated on the test set.

### 2.9 Evaluation

We evaluate all methods on the four different synthetic datasets and the ADNI dataset. Characteristics of all datasets are summarized in Table 2.

**Table 2:** Summary of dataset characteristics.

**Dataset**	**Subjects**	**Censoring percentage**	**Mean event time**	**Mean number of visits**
Scenario 1	1,000 (train)	33%	5.1 ± 3.0	10.1 ± 5.9
Scenario 2	1,000 (train)	33%	5.1 ± 3.0	10.2 ± 6.0
Scenario 3	1,000 (train)	42%	5.0 ± 3.2	10.0 ± 6.3
Scenario 4	1,000 (train)	48%	5.2 ± 3.3	10.4 ± 6.6
ADNI	1,390	83%	3.5 ± 2.4	7.2 ± 3.9

To ensure a reliable estimate for generalization performance, we use a separate set of subjects for evaluation, that will not be used during model training. For the simulation scenarios we create 10 different training sets with 1,000 subjects and generate a large set of 3,000 different subjects for evaluation. For the ADNI data we use 10-fold cross validation, where the subjects are divided among ten sets and we repeatedly use one set for evaluation and all others for training.

For each dataset we choose four landmark points *l* at which we evaluate the predictions of the conditional survival probability. We landmark the test dataset at time *l*, by removing subjects with *T*_*i*_ < = *l* and removing measurements obtained after time *l*. The fitted models are then used to predict the survival probability at several time points *t*>*l*.

At each time *t* we calculate the predicted risk $\hat{R}\left(t|Y_{i}^{\left(l\right)}\right)$ of a subject, conditional on survival until *l*, using


(18) $$\begin{array}{l} \hat{R}\left(t|Y_{i}^{\left(l\right)}\right)=1-\frac{Ŝ\left(t|Y_{i}^{\left(l\right)}\right)}{Ŝ\left(l|Y_{i}^{\left(l\right)}\right)}. \end{array}$$

To adjust for censoring we calculate the Inverse Probability of Censoring Weighting (IPCW). For this we use the training data to obtain the Kaplan-Meier estimate of the censoring distribution:


(19) $$\begin{array}{l} Ĝ\left(t\right)=\underset{j:t_{j}<=t}{\prod }\left(1-\frac{\sum_{i\in \text{train}}\left(1-\delta_{i}\right)\cdot I\left(T_{i}=t_{j}\right)}{\sum_{i\in \text{train}}I\left(T_{i}>=t_{j}\right)}\right) \end{array}$$

To assess the discrimination performance of models, we calculate the IPCW time-dependent Area Under the Curve (tdAUC) (36). In Blanche et al. (37), it is shown that this is a proper metric for predicting the risk of an event *t* years in the future. The tdAUC compares all pairs of subject (*i, j*) where subject *i* has the event of interest at or before time *t* and subject *j* is still event free at *t*. When the predicted risk of subject *i* at time *t* is higher, as expected, the score increases. The complete formula is given by


(20) $$\begin{array}{l} \text{ tdAUC}C_{\text{IPCW}}\left(t\right)\\ =\frac{\sum_{i=1}^{N}\sum_{j=1}^{N}\delta_{i}\cdot I\left(T_{i}\leq t\right)I\left(T_{j}>t\right)Ĝ\left(T_{i}\right)I\left(\hat{R}\left(t|Y_{i}^{\left(l\right)}\right)>\hat{R}\left(t|Y_{j}^{\left(l\right)}\right)\right)}{\left(\sum_{i=1}^{N}I\left(T_{i}>t\right)\right)\left(\sum_{i=1}^{N}\delta_{i}\cdot I\left(T_{i}\leq t\right)Ĝ\left(T_{i}\right)\right)}. \end{array}$$

In addition, we measure the error of the models using the IPCW Brier Score. This metric is an estimation of the Mean Squared Error when the true underlying probabilities are unknown, but only binary outcomes are available and is given by


(21) $$\begin{array}{l} BS_{\text{IPCW}}\left(t\right)=\frac{1}{N}\overset{N}{\underset{i=1}{\sum }}\delta_{i}\cdot I\left(T_{i}<=t\right)\frac{{\left(1-\hat{R}\left(t|Y_{i}^{\left(l\right)}\right)\right)}^{2}}{Ĝ\left(T_{i}\right)}\\ \;+I\left(T_{i}>t\right)\frac{{\left(0-\hat{R}\left(t|Y_{i}^{\left(l\right)}\right)\right)}^{2}}{Ĝ\left(t\right)}. \end{array}$$

For the simulated datasets, we do have access to underlying survival probabilities, which allows us to calculate the mean squared error using the following equation


(22) $$\begin{array}{l} MSE\left(t\right)=\frac{1}{N}\overset{N}{\underset{i=1}{\sum }}{\left(R\left(t|Y_{i}^{\left(l\right)}\right)-\hat{R}\left(t|Y_{i}^{\left(l\right)}\right)\right)}^{2}. \end{array}$$

**Figure 1:**
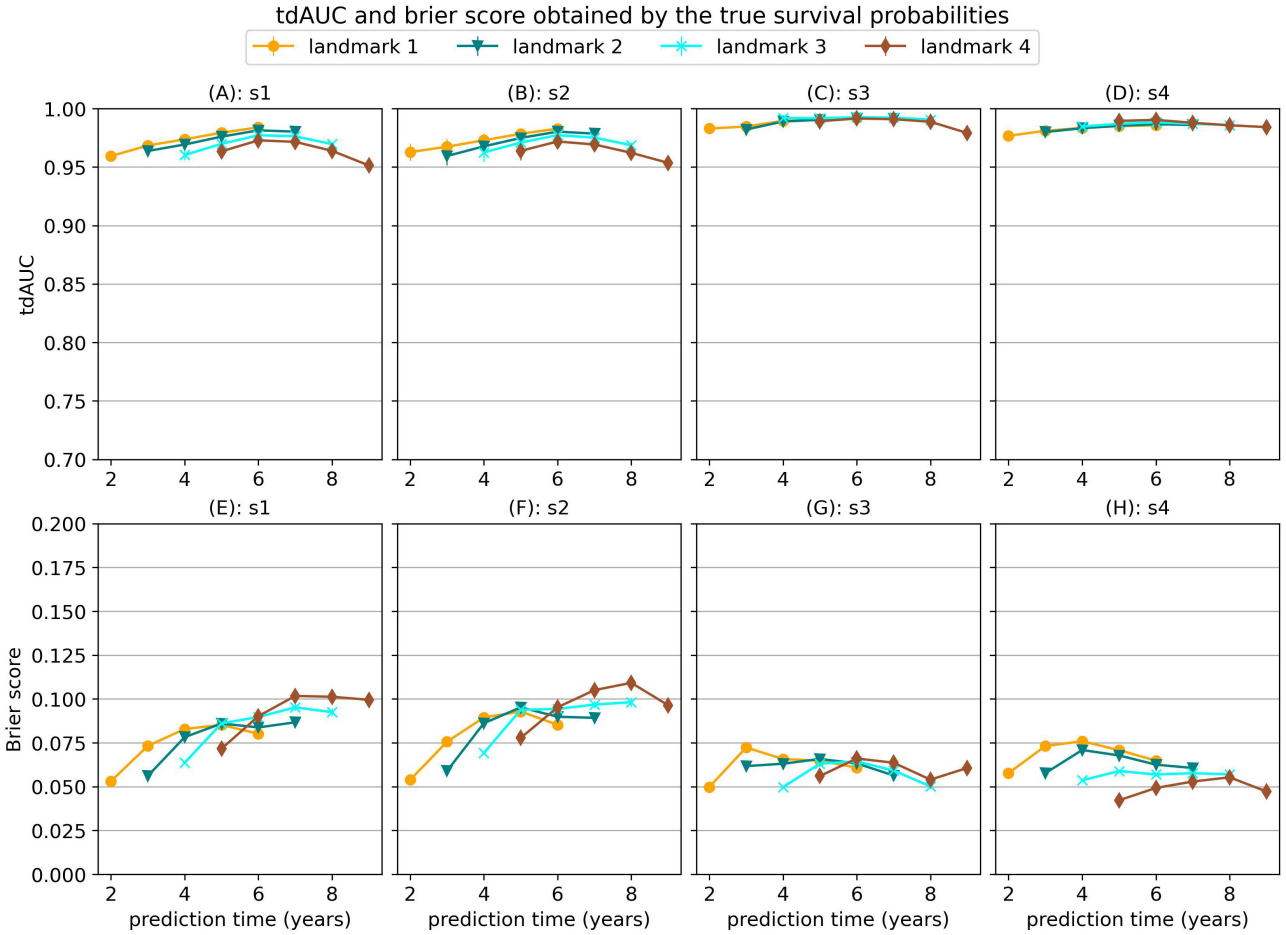
tdAUC and brier score results obtained by predicting the true survival probabilities for each simulation scenario.

When a model would perfectly predict the true survival probabilities this would results in a MSE of 0. However, this would not necessarily result in perfect scores for the tdAUC and brier score metrics. In Figure 1 we show the tdAUC and brier score results obtained by predicting the true survival probabilities for each simulation scenario.

## 3 Results

### 3.1 Results on synthetic experiments

First, we will investigate the behaviour of the different models on the synthetic datasets. Due to the large amount of models, datasets, landmarking methods and metrics, we will focus on the most salient observations. Additional figures can be found in the Supplementary material or in the code repository (https://github.com/Wieske/DSA_comparison).

#### 3.1.1 Effect of dependency structure

**Figure 2:**
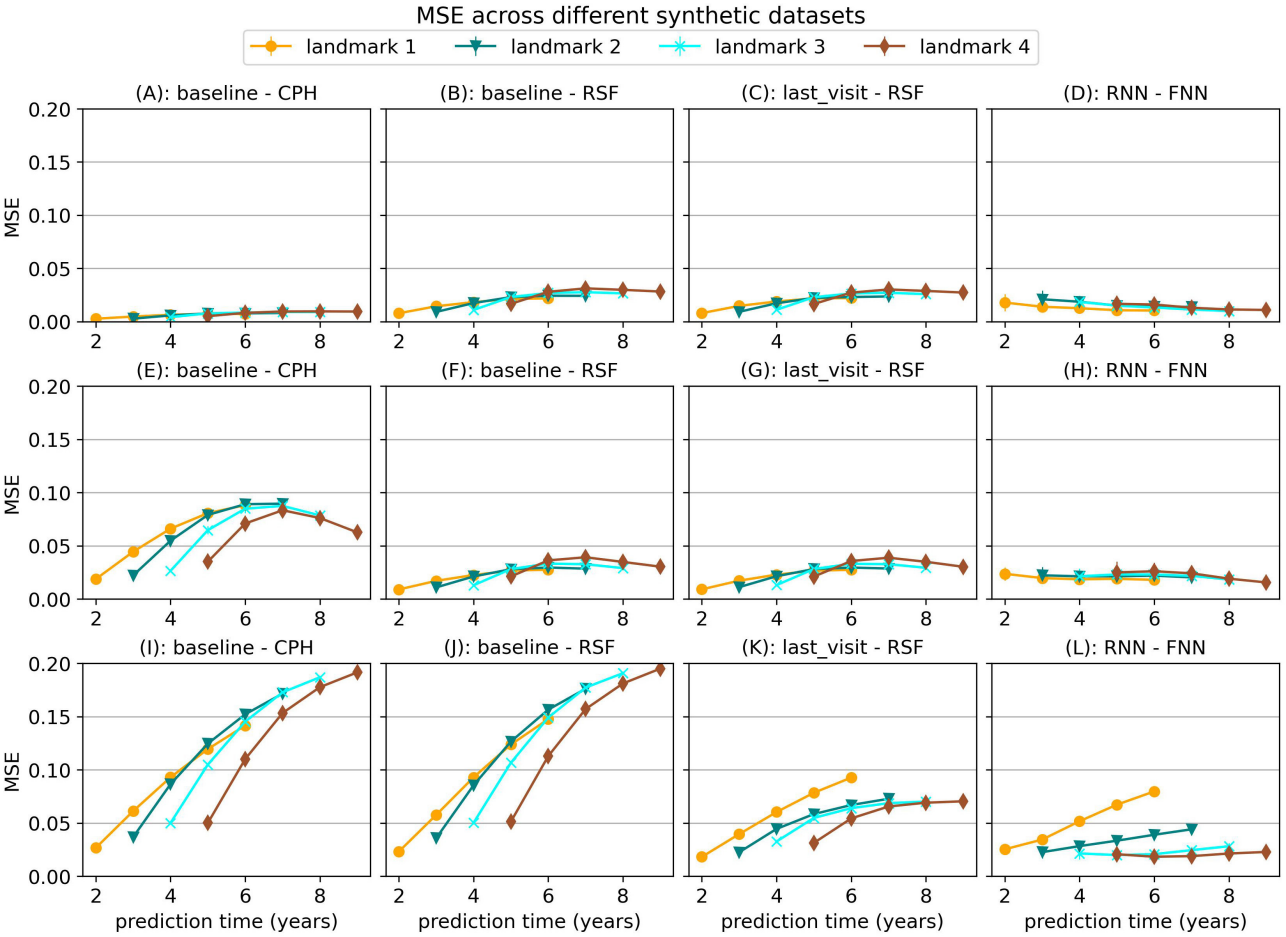
Mean Squared Error (MSE) for a selection of models across the first three simulation scenarios. The markers represent the mean of 10 results, with error bars illustrating the standard deviation. The top row shows the results for scenario 1 (baseline), the middle row for scenario 2 (with interaction term) and the bottom row for scenario 3 (longitudinal). For MSE lower is better.

**Figure 3:**
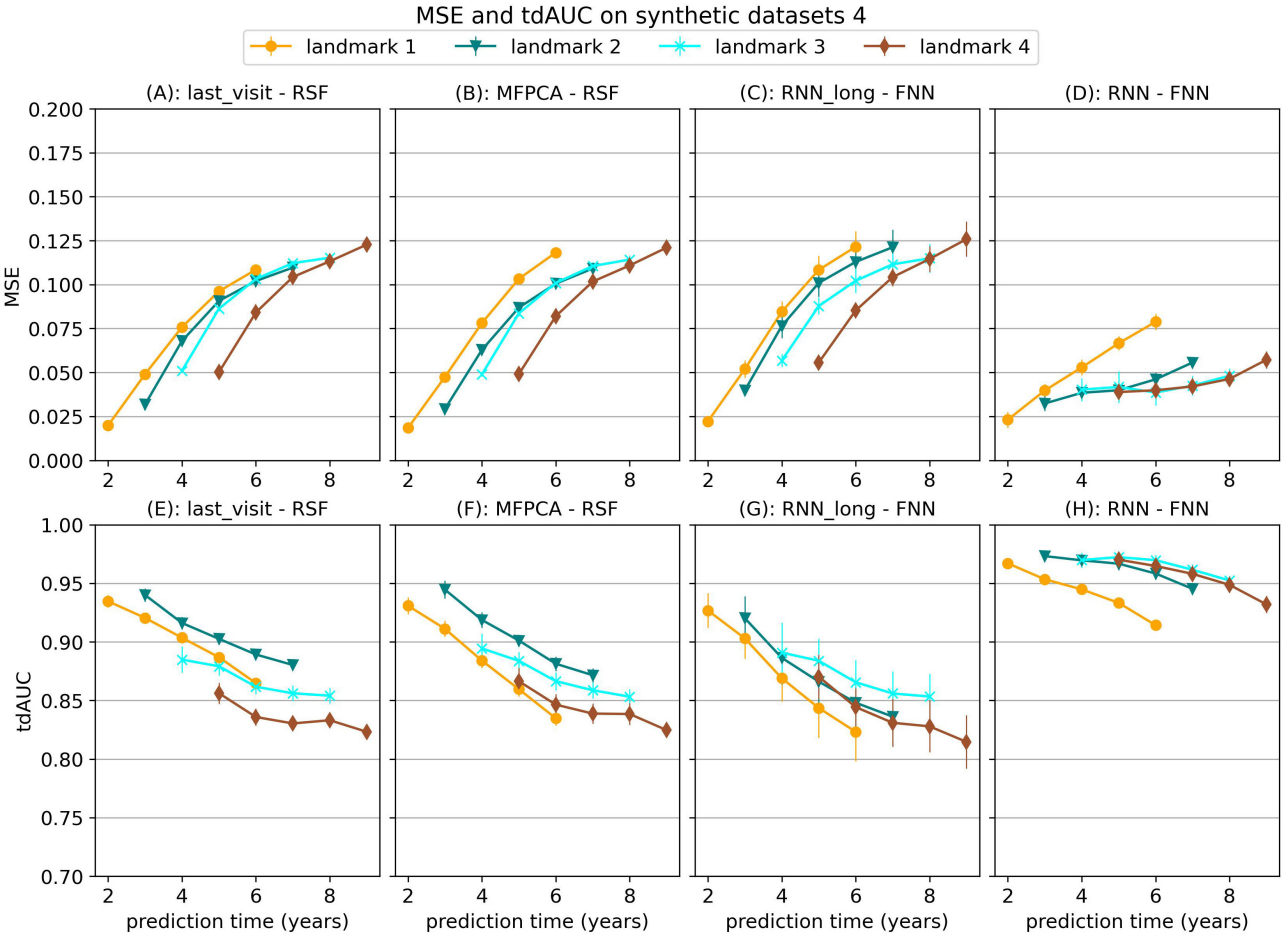
Mean Squared Error (MSE) and tdAUC for a selection of models for synthetic scenario 4 (delayed effect). The markers represent the mean of 10 results, with error bars illustrating the standard deviation. For MSE lower is better and for tdAUC higher is better.

First we find that the choice of model is very dependent on the type of relationships that exist in the data. In Figure 2 we show the MSE for several models on the first three synthetic datasets, which were all trained with the strict landmarking method. In Figure 3 we show the results for a different selection of models on scenario 4.

For simulation scenario 1, where the longitudinal trajectories of the subjects can be extrapolated from baseline data, we see that a Cox Proportional Hazard (CPH) model that just uses baseline data gives almost perfect results. When we add an interaction term in the simulation (scenario 2) that is not specified in the CPH model we can see the advantage of models such as Random Survival Forest. This survival model has more flexibility and can therefore learn this interaction term even when it is not explicitly provided to the model.

In simulation scenario 3 we introduce different slopes for different subjects, which means it is no longer possible to extrapolate the trajectories from baseline data alone. As a result, the baseline model can no longer provide enough information and the MSE for this model becomes larger when more time passes. In this case, using the last available measurement instead of the baseline model already results in a significant decrease in error. Using a neural network method (RNN-FNN) results in even more improvement, especially at later landmark times, where more data points are available to estimate the future trajectory.

In the last scenario we add a randomly sampled covariate with a delayed effect on the hazard. For this covariate the future trajectory cannot be accurately predicted, but the effect can be predicted using historical values. The results in Figure 3 show that the RNN-FNN model outperforms other approaches for this scenario. This model is trained on longitudinal and survival prediction simultaneously, which likely makes it possibly to detect this effect.

#### 3.1.2 Landmarking methods

**Figure 4:**
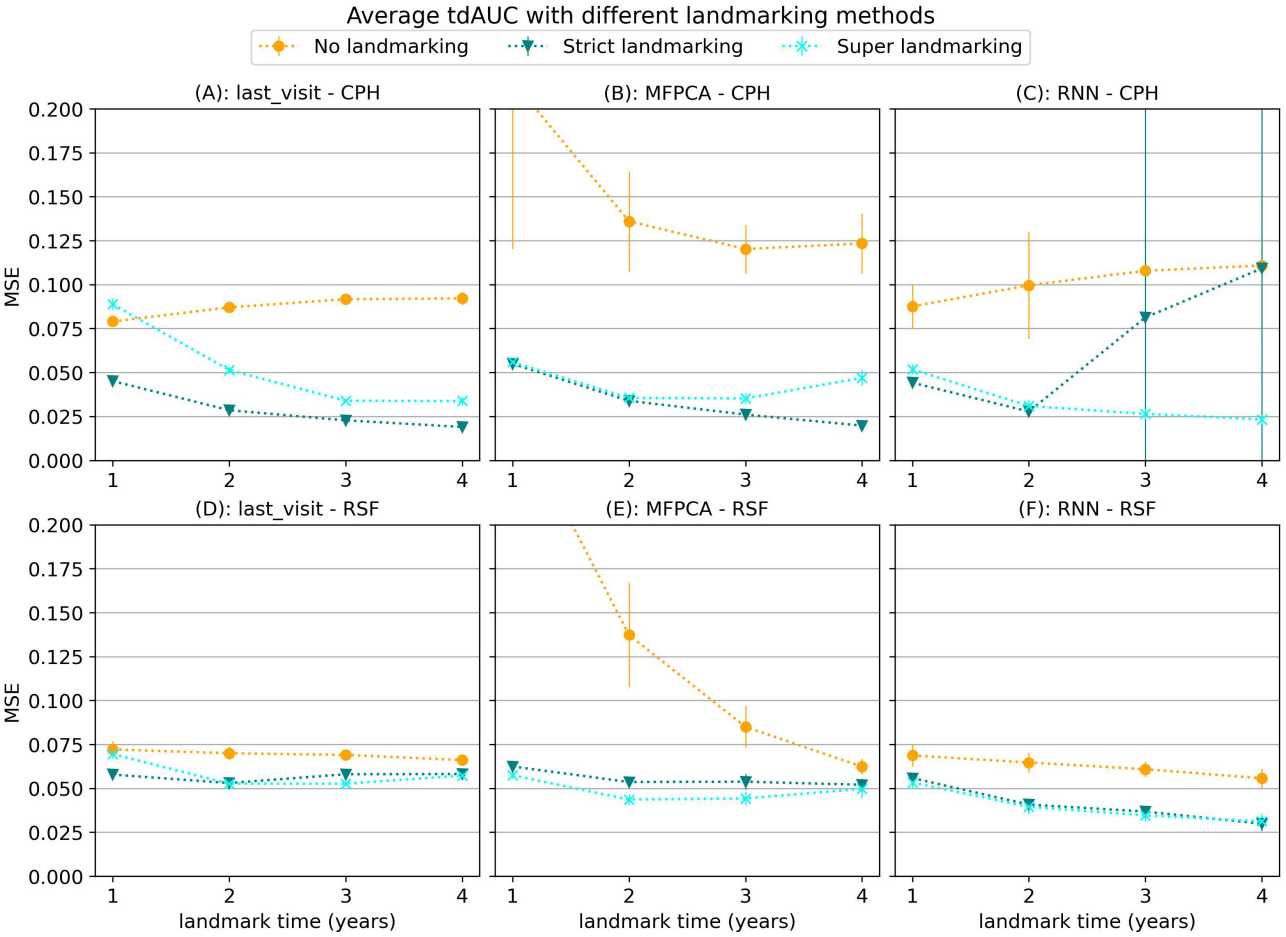
Average tdAUC for several model combinations using different landmarking methods during training. The markers represent the mean of 10 results, with error bars illustrating the standard deviation. For the tdAUC higher is better.

Next we will focus on the results for different landmarking methods, for which we will use synthetic data scenario 3. In Figure 4 we show the average MSE per landmark time for different landmarking methods. Although the results vary based on which models are used, in general using no landmarking results in the worst performance. For the MFPCA model not using landmarking resulted in a large error, especially for early landmarks. This error decreases for later landmarks, possibly because by then the trajectories used for evaluation are longer, making them more similar to the complete longitudinal trajectories in the training set. The difference between strict landmarking and super landmarking is usually smaller and differs per model. For the CPH model the strict landmarking method is usually superior, except for the combination with the RNN model, where some of the runs did not properly converge.

#### 3.1.3 Neural network training strategies

**Figure 5:**
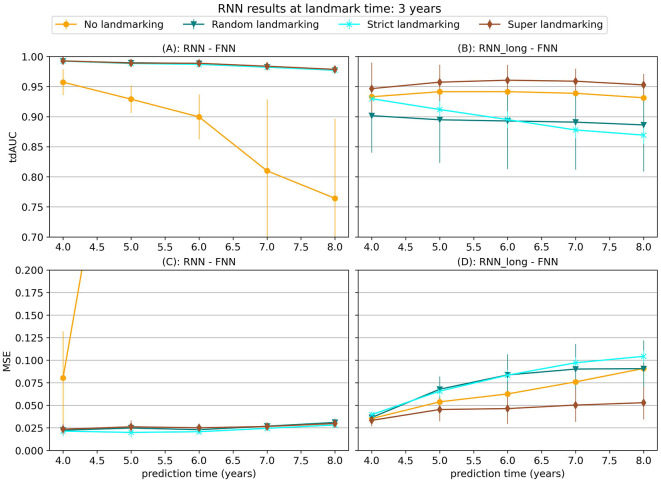
tdAUC and MSE results for the RNN-FNN and RNN_long-FNN models using different landmarking strategies with data from synthetic scenario 3 at a landmark time of 3 years. The markers represent the mean of 10 results, with error bars illustrating the standard deviation. For the tdAUC higher is better and for the MSE lower is better.

In Figure 5 results are shown for the neural network methods, consisting of a recurrent neural network (RNN), followed by a fully-connected neural network (FNN). For this model two combinations are trained, one where the RNN is only trained on a longitudinal loss (RNN_long-FNN) and one where the RNN is first trained on a combination loss and afterwards the entire model is trained on a survival loss (negative log likelihood) (RNN-FNN).

When one of the landmarking methods is applied during training the RNN-FNN model has the best performance. However, without landmarking the RNN-FNN model has a very high error, while the RNN_long-FNN model still gives decent results. This is likely because the RNN-FNN model is overfitting on information in the complete longitudinal trajectories that will not be available during evaluation, as also explained in Section 2.3.1. During training the model can determine the survival probability of subjects using values of the covariates right before the event, so it is not necessary to try to estimate future values for the covariates to make a good prediction. During evaluation these values are not always available due to landmarking of the data, which causes the high errors. By only using longitudinal loss the RNN_long model is forced to learn features that can be used to predict the future trajectory, which are still informative when the data is landmarked during evaluation. When some form of landmarking is used during training, the RNN-FNN model does not have this problem and the bigger focus on the survival loss seems to become an advantage.

### 3.2 Results on real world data

**Figure 6:**
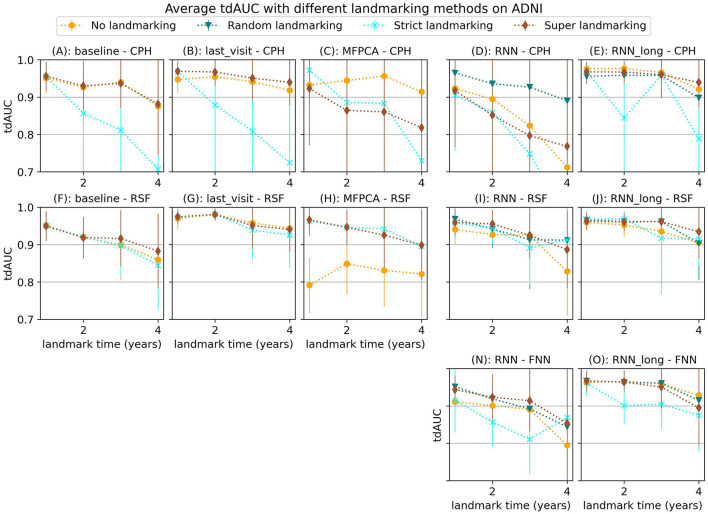
Average tdAUC for all considered model combinations on the ADNI dataset with different landmarking methods. The markers represent the mean of 10 results, with error bars illustrating the standard deviation. For the tdAUC higher is better.

**Figure 7:**
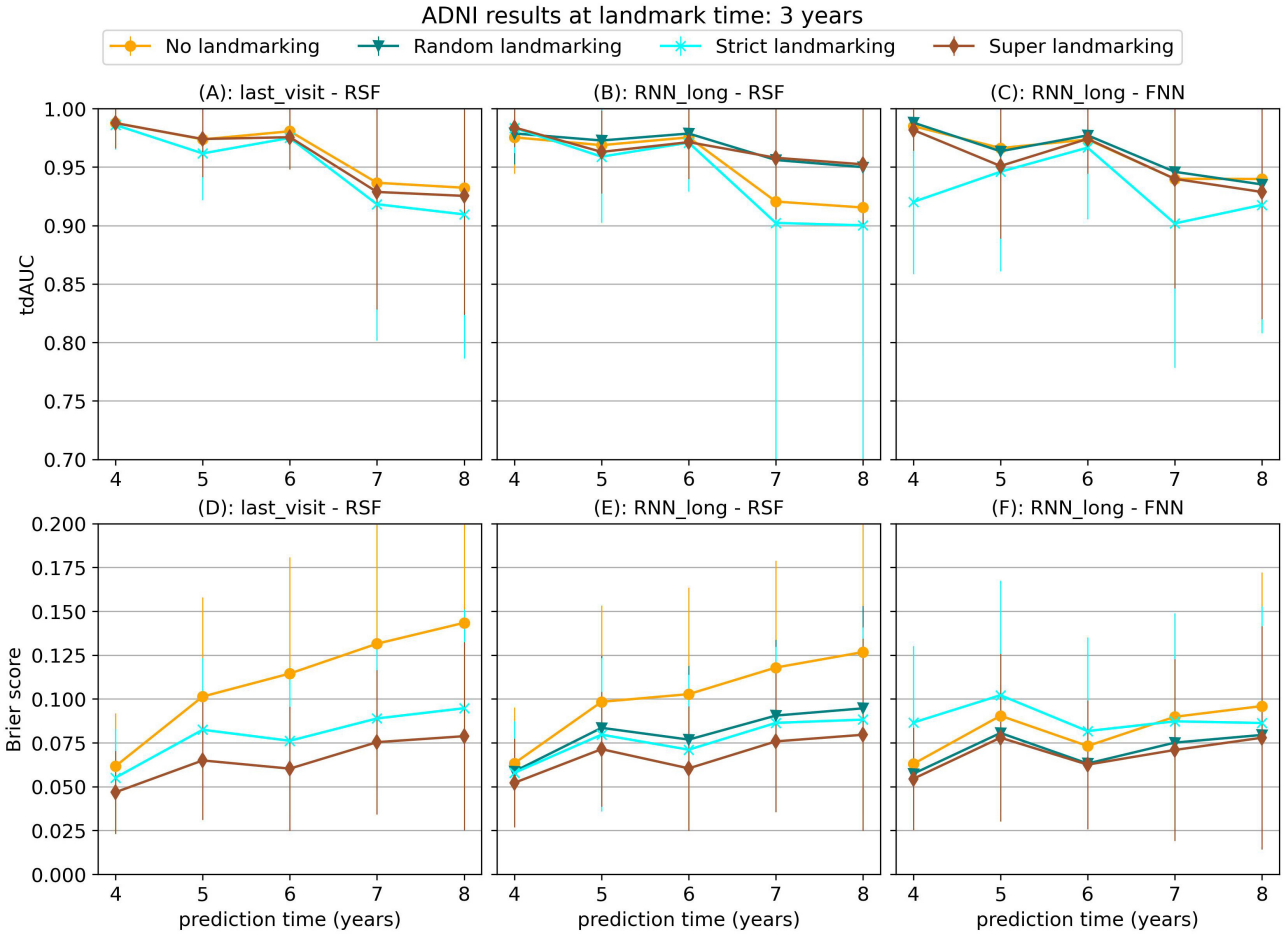
tdAUC and brier score for several models on the ADNI dataset at a landmark time of 3 years, using different landmarking methods. The markers represent the mean of 10 results, with error bars illustrating the standard deviation. For the tdAUC higher is better and for the brier score lower is better.

Our experiments on the ADNI dataset show less conclusive results. Figure 6 displays the average tdAUC for each combination of model and landmarking method. In Figure 7 we show the tdAUC and brier score over time for several model combinations at a landmark time of 3 years.

For this dataset, super and random landmarking often outperform both strict landmarking and no landmarking. The inferior results of the strict landmarking method may be attributed to specific characteristics of the ADNI dataset. The ADNI study was conducted in multiple phases, each recruiting new participants while continuing to follow subjects from previous phases. Participants who entered during later phases had fewer visits before the study concluded. Therefore excluding subjects that have had the event results in a rapid decline in the number of subjects available for training over time. Specifically, after 1 year 84% of subjects are still at risk (1,161), after 2 year 61% (842 subjects), after 3 years 42% (583 subjects), and after 4 years only 22% (301 subjects). The results of the RSF model only decrease slightly at later landmark times, but for the CPH and FNN models, the smaller sample size of strict landmarking resulted in poor performance. For the CPH model, this might be caused by convergence or numerical issues as a result of the larger number of variables without regularization. The neural network models might have overfit on the smaller training set.

Among the different survival models tested, the Random Survival Forest (RSF) model frequently produced strong results, demonstrating its ability to handle complex survival data with many features. RSF was also the most stable across landmarking methods, showing similar performance regardless of the chosen strategy. In contrast, the CPH model often performed poorly, which may be due to the large number of features and the lack of regularization, leading to numerical or convergence issues in some runs. The FNN model resulted in similar or worse performance compared to the RSF.

Among the longitudinal models, the RNN_long model demonstrated good performance but did not improve upon the simpler approach of using only the last visit for prediction. Unlike the results observed for synthetic data in Section 3.1.3, incorporating additional survival information into the RNN only diminished the performance. These results suggest that for this dataset, these more complex strategies are either unnecessary or need further refinement to leverage their full potential.

### 3.3 Sensitivity to hyperparameter choices

**Figure 8:**
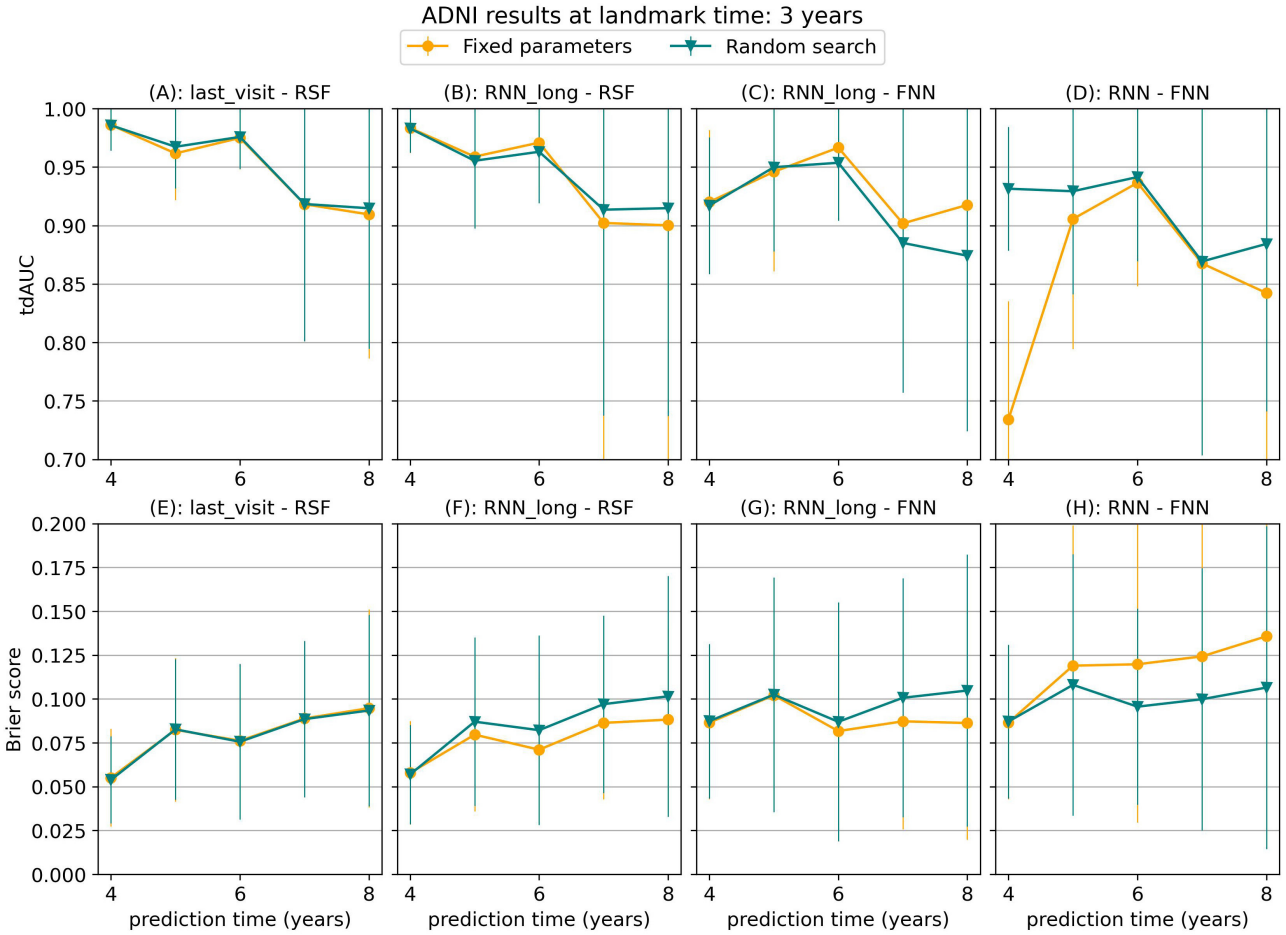
tdAUC and brier score obtained using fixed parameters or a random hyperparameter search for several models on the ADNI dataset at a landmark time of 3 years, using the strict landmarking method. The markers represent the mean of 10 results, with error bars illustrating the standard deviation. For the tdAUC higher is better and for the brier score lower is better.

To assess the sensitivity of our results to the chosen hyperparameters, we performed a random hyperparameter search on the ADNI dataset for a landmark time of 3 years, using the strict landmarking method. Figure 8 compares the results of this random search to the results obtained with the fixed parameter set. For most models, the results were comparable between the two approaches, with the fixed hyperparameter set even outperforming random search at several points. For the RNN-FNN model, the random search led to performance improvements, especially at points where the original results were poor compared to other models. However, even with these improved results, the RNN-FNN model did not surpass the performance of the last_visit-RSF model. This suggests that the qualitative conclusions drawn from our experiments do not strongly depend on our choice of fixed hyperparameters.

### 3.4 Computational requirements

**Figure 9:**
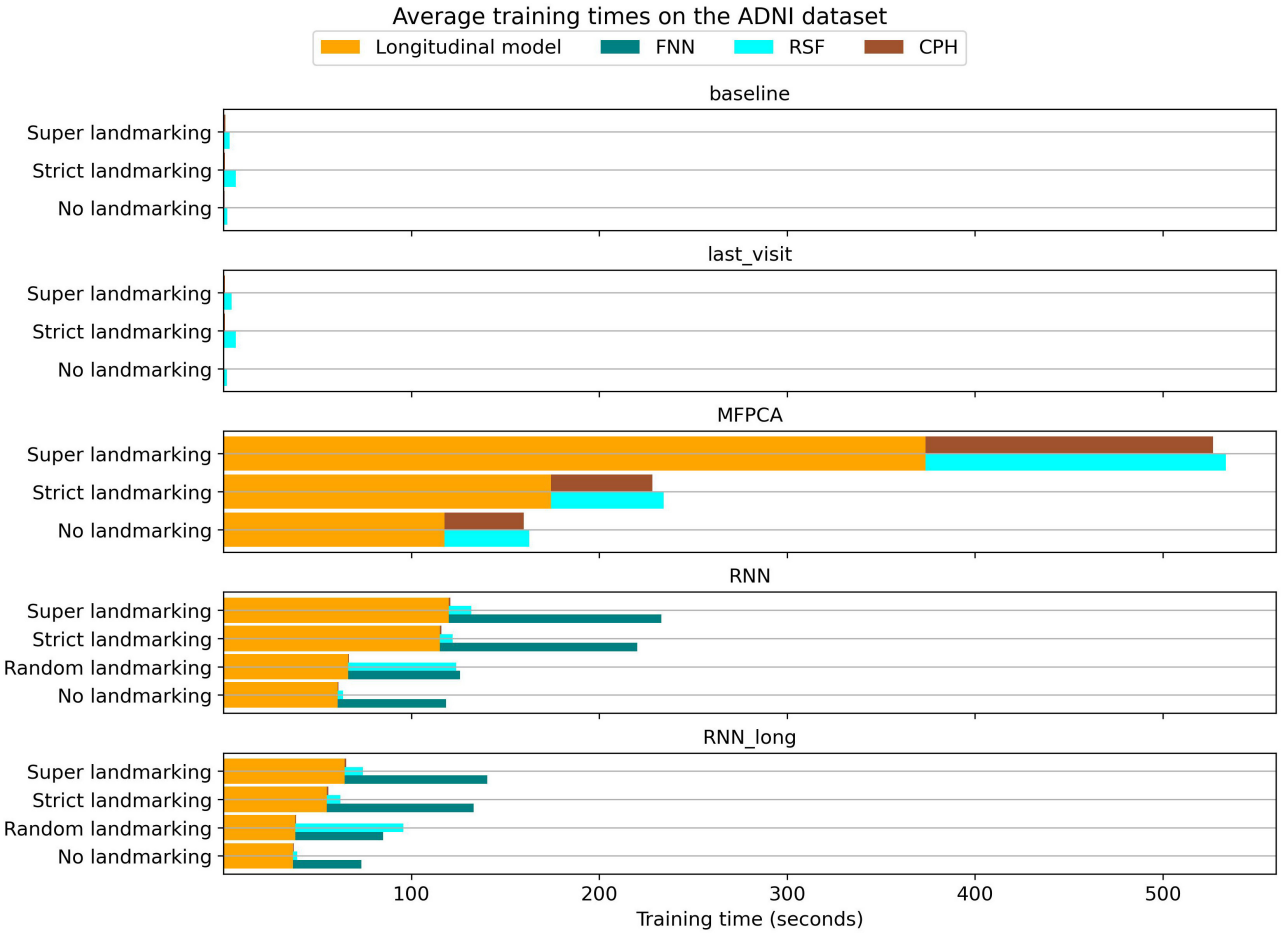
Training times for different models and landmarking methods on the ADNI dataset.

An additional consideration in model selection is the computational efficiency of the chosen method. We recorded the training times for each model on the ADNI dataset, with the average results shown in Figure 9. While all methods were trained using similar computational resources, the neural network models benefited from GPU acceleration, reducing their training times.

The baseline and last_visit benchmarks were substantially faster than other methods, as these benchmarks only required training the survival model. In contrast, the MFPCA method was notably slower, both in training the longitudinal model and in generating longitudinal encodings for subsequent survival modeling. This higher inference cost extended the training time for the survival models.

Among landmarking strategies, no landmarking and random landmarking were the most computationally efficient. Super and strict landmarking, on the other hand, resulted in longer training times due to their larger combined training datasets. Specifically, super landmarking trains a single model on the entire expanded dataset, whereas strict landmarking involves training separate models on smaller subsets of the data. For neural network methods, these two landmarking strategies yielded comparable training times. However, for MFPCA and RSF, super landmarking was slower because these models required significantly more time to handle the larger dataset.

## 4 Discussion

This study explored various landmarking strategies and model choices for dynamically predicting survival outcomes, highlighting the importance of these decisions in different contexts. Our findings suggest that some form of landmarking is often beneficial, but the optimal strategy can depend heavily on the specific characteristics of the dataset and the model being used.

In the simulation experiments, strict landmarking was often the best-performing method. On the ADNI data, on the other hand, it often performed poorly. This may be because, while this method has a low bias in theory, the exclusion of subjects can result in higher variance. Therefore, settings with limited data can benefit from different landmarking approaches, such as the super or random landmarking method. These methods resulted in good performance for most models with synthetic data as well as ADNI.

It should be noted that our focus has been on observational datasets, where the baseline time of a subject usually has no special meaning. A strict landmarking method may be more beneficial in an intervention setting because the trajectories of the subjects are potentially better aligned at the start of the intervention. In this setting, the considered landmark times are expected to be at more similar points in all trajectories. As a result, measurements between subjects can be more readily compared from time point to time point, which will typically lead to improved performance.

Our experiments, conducted on datasets with varying characteristics, demonstrate that the optimal longitudinal model can vary depending on the specific scenario. In simpler settings, where longitudinal trajectories show little variation between subjects, classical survival analysis methods fit with baseline data are sufficient. However, this approach falls short as soon as longitudinal trajectories exhibit more variation within subjects. In the more complex simulation scenarios as well as the real-world setting, several methods exceeded the performance of the baseline benchmark. The RNN model demonstrated strong performance across all synthetic datasets, with its advantages particularly evident in the final scenario. In this case, it appeared to be the only model capable of accurately capturing the effect of the additional covariate. This highlights the importance of the ability to integrate longitudinal and survival information during training, which can be crucial for modeling certain complex relationships. In the scenarios we considered, the MFPCA model never emerged as the best-performing approach. On the ADNI dataset, the last visit benchmark delivered the best results, emphasizing that incorporating the entire longitudinal data is not always necessary for optimal performance.

Among the studied survival models, one clear takeaway is the robustness and versatility of the Random Survival Forest (RSF). Across most longitudinal models and landmarking methods, RSF consistently produced stable results and strong performance. Its ability to handle non-linearities and its ease of implementation make it an attractive choice for many applications. In the synthetic data scenario where the model assumptions were correctly specified, the Cox Proportional Hazards (CPH) model delivered the best results. This suggests that CPH may be a suitable choice when the relationships between covariates and event probabilities are well understood and align with the model's underlying assumptions.

While neural network-based strategies did show some benefits when survival information was incorporated into the longitudinal model, this improvement was primarily observed in synthetic data. When applied to the ADNI dataset, these strategies failed to outperform simpler benchmarks, indicating that the value of complex models depends strongly on the characteristics of the data. The ADNI dataset is characterized by a high percentage of censored observations and a lower average number of visits per subject compared to our synthetic datasets, as shown in Table 2. This combination poses challenges for identifying complex relationships between covariates and event probabilities. The limited frequency of longitudinal measurements reduces the amount of information available to capture temporal patterns, while the high censoring rate further restricts the effective sample size for modeling event occurrences. These factors likely contribute to the difficulty in detecting nuanced interactions and may limit the performance of more complex models on this dataset.

Our results suggest that special care should be taken when benchmarking new methods. Not only should they be compared to simple variations of the proposed technique, which often is the only experiment carried out, but a comparison should also be made with rather different approaches. Our results on ADNI show that it is sensible to include a simple method like last_visit that corresponds to the traditional landmarking strategy, as described in Section 2.2.

In our study, we used a fixed set of hyperparameters across all models to ensure fair comparisons, but we recognize this as a potential limitation. To address this, we conducted an additional experiment involving a random hyperparameter search in a limited setting. This experiment demonstrated improved performance for some neural network methods, particularly in scenarios where their initial results were suboptimal. However, these improvements did not alter the overarching conclusions of our study. While optimizing hyperparameters, especially for complex models like neural networks, can enhance predictive performance, it also introduces a trade-off. High sensitivity to hyperparameters can reduce model robustness and make predictions less reliable. Therefore, while hyperparameter tuning has the potential to refine model outcomes, it must be approached carefully to balance improved performance with the need for robustness and generalizability.

In conclusion, our results highlight the importance of carefully considering both landmarking strategies and model choices when dynamically predicting survival outcomes. Across most scenarios considered, incorporating landmarking during training proves beneficial. Strict landmarking performs well when enough data is available, while the super and random landmarking approaches offer excellent performance in the ADNI setting, with limited data. Among the different model combinations, the random survival forest fitted on the last available measurement consistently provides strong results, making it a reliable choice in many cases. Neural network methods show some potential for improvement when sufficient data and informative longitudinal trajectories are present. Ultimately, the optimal combination depends on factors such as the complexity of the data relationships, the available data, and the intended application of the model. Therefore, understanding the trade-offs between these choices is essential for successful dynamic survival analysis.

## Data Availability

The simulated datasets for this study can be found in the Github repository at https://github.com/Wieske/DSA_comparison. Access to the ADNI data can be requested from the website: https://adni.loni.usc.edu/.

## Footnotes

^1^ https://tadpole.grand-challenge.org

## References

[1] Lee C Yoon J Schaar Mvd. Dynamic-DeepHit: a deep learning approach for dynamic survival analysis with competing risks based on longitudinal data. IEEE Trans Biomed Eng. (2020) 67:122–33. 10.1109/TBME.2019.2909027 30951460

[2] Jarrett D Yoon J Schaar Mvd. Dynamic prediction in clinical survival analysis using temporal convolutional networks. IEEE J Biomed Health Inform. (2020) 24:424–36. 10.1109/JBHI.2019.2929264 31331898

[3] Tanner KT Sharples LD Daniel RM Keogh RH. Dynamic survival prediction combining landmarking with a machine learning ensemble: methodology and empirical comparison. J R Stat Soc. (2021) 184:3–30. 10.1111/rssa.12611

[4] Lin J Luo S. Deep learning for the dynamic prediction of multivariate longitudinal and survival data. Stat Med. (2022) 41:2894–2907. 10.1002/sim.9392 35347750 PMC9232978

[5] Chowdhary N Barbui C Anstey KJ Kivipelto M Barbera M Peters R. Reducing the risk of cognitive decline and dementia: WHO recommendations. Front Neurol. (2022) 12:765584. 10.3389/fneur.2021.765584 35082745 PMC8784726

[6] Livingston G Huntley J Liu KY Costafreda SG Selbk G Alladi S. Dementia prevention, intervention, and care: 2024 report of the Lancet standing Commission. Lancet. (2024) 404:572–628. 10.1016/S0140-6736(24)01296-0 39096926

[7] Rizopoulos D Molenberghs G Lesaffre EMEH. Dynamic predictions with time-dependent covariates in survival analysis using joint modeling and landmarking. Biom J. (2017) 59:1261–76. 10.1002/bimj.201600238 28792080

[8] Suresh K Taylor JMG Spratt DE Daignault S Tsodikov A. Comparison of joint modeling and landmarking for dynamic prediction under an illness-death model. Biom J. (2017) 59:1277–300. 10.1002/bimj.201600235 28508545 PMC5957493

[9] Van Houwelingen HC. Dynamic prediction by landmarking in event history analysis. Scand J Stat. (2007) 34:70–85. 10.1111/j.1467-9469.2006.00529.x

[10] Tsiatis AA Davidian M. Joint modeling of longitudinal and time-to-event data: an overview. Stat Sin. (2004) 14:809–34. Available at: http://www.jstor.org/stable/24307417

[11] Rizopoulos D. Joint Models for Longitudinal and Time-to-Event Data: With Applications in R. London: CRC Press. (2012). 10.1201/b12208

[12] Hickey GL Philipson P Jorgensen A Kolamunnage-Dona R. Joint modelling of time-to-event and multivariate longitudinal outcomes: recent developments and issues. BMC Med Res Methodol. (2016) 16:117. 10.1186/s12874-016-0212-5 27604810 PMC5015261

[13] Mauff K Steyerberg E Kardys I Boersma E Rizopoulos D. Joint models with multiple longitudinal outcomes and a time-to-event outcome: a corrected two-stage approach. Stat Comput. (2020) 30:999–1014. 10.1007/s11222-020-09927-9

[14] Putter H van Houwelingen HC. Landmarking 2.0: bridging the gap between joint models and landmarking. Stat Med. (2022) 41:1901–17. 10.1002/sim.9336 35098578 PMC9304216

[15] Lin J Li K Luo S. Functional survival forests for multivariate longitudinal outcomes: dynamic prediction of Alzheimer's disease progression. Stat Methods Med Res. (2021) 30:99–111. 10.1177/0962280220941532 32726189 PMC7855476

[16] Gomon D Putter H Fiocco M Signorelli M. Dynamic prediction of survival using multivariate functional principal component analysis: a strict landmarking approach. Stat Methods Med Res. (2024) 33:256–72. 10.1177/09622802231224631 38196243 PMC10928955

[17] Li K Luo S. Dynamic prediction of Alzheimer's disease progression using features of multiple longitudinal outcomes and time-to-event data. Stat Med. (2019) 38:4804–18. 10.1002/sim.8334 31386218 PMC6800781

[18] Van Houwelingen H Putter H. Dynamic Prediction in Clinical Survival Analysis. London: CRC Press. (2011). 10.1201/b11311

[19] Shorten C Khoshgoftaar TM. A survey on image data augmentation for deep learning. J Big Data. (2019) 6:60. 10.1186/s40537-019-0197-0 PMC8287113 34306963

[20] Ramsay J Silverman BW. Functional Data Analysis. Cham: Springer. (2005). 10.1007/b98888

[21] Yao F Müller HG Wang JL. Functional data analysis for sparse longitudinal data. J Am Stat Assoc. (2005) 100:577–90. 10.1198/01621450400000174512611515

[22] Happ C Greven S. Multivariate functional principal component analysis for data observed on different (dimensional) domains. J Am Stat Assoc. (2018) 113:649–59. 10.1080/01621459.2016.127311529051679

[23] Elman JL. Finding structure in time. Cogn Sci. (1990) 14:179–211. 10.1016/0364-0213(90)90002-E

[24] Hochreiter S. Long short-term memory. Neur Comput. (1997) 9:1735–1780. 10.1162/neco.1997.9.8.1735 9377276

[25] Zeng L Zhang J Chen W Ding Y. tdCoxSNN: time-dependent Cox survival neural network for continuous-time dynamic prediction. J R Stat Soc C. (2024) 74:187–203. 10.1093/jrsssc/qlae051 39807175 PMC11725344

[26] Cox DR. Regression models and life-tables. J R Stat Soc. (1972) 34:187–202. 10.1111/j.2517-6161.1972.tb00899.x

[27] Efron B. The efficiency of Cox's likelihood function for censored data. J Am Stat Assoc. (1977) 72:557–65. 10.1080/01621459.1977.1048061337016341

[28] Breslow N. Covariance analysis of censored survival data. Biometrics. (1974) 30:89-99. 10.2307/2529620 4813387

[29] Ishwaran H Kogalur UB Blackstone EH Lauer MS. Random survival forests. Ann Appl Stat. (2008) 2:841–60. 10.1214/08-AOAS169

[30] Zadeh SG Schmid M. Bias in cross-entropy-based training of deep survival networks. IEEE Trans Pattern Anal Mach Intell. (2021) 43:3126–37. 10.1109/TPAMI.2020.2979450 32149626

[31] Marinescu RV Oxtoby NP Young AL Bron EE Toga AW Weiner MW. TADPOLE challenge: prediction of longitudinal evolution in Alzheimer's disease. ArXiv:1805.03909. (2018).

[32] Srivastava N Hinton G Krizhevsky A Sutskever I Salakhutdinov R. Dropout: a simple way to prevent neural networks from overfitting. J Mach Learn Res. (2014) 15:1929–58. Available at: http://jmlr.org/papers/v15/srivastava14a.html33259321

[33] Kingma DP. Adam: a method for stochastic optimization. arXiv preprint arXiv:1412.6980. (2014).

[34] Zhang G Wang C Xu B Grosse R. Three mechanisms of weight decay regularization. arXiv preprint arXiv:1810.12281. (2018).

[35] Bergstra J Bengio Y. Random search for hyper-parameter optimization. J Mach Learn Res. (2012) 13:281–305. Available at: http://jmlr.org/papers/v13/bergstra12a.html

[36] Uno H Cai T Tian L Wei LJ. Evaluating prediction rules for t-year survivors with censored regression models. J Am Stat Assoc. (2007) 102:527–37. 10.1198/01621450700000014912611515

[37] Blanche P Kattan MW Gerds TA. The c-index is not proper for the evaluation of t-year predicted risks. Biostatistics. (2019) 20:347–57. 10.1093/biostatistics/kxy006 29462286

