# Supplementary material for: A comparative study of methods for dynamic survival analysis
Source: Front Neurol. 2025 Feb 18;16:1504535. doi: 10.3389/fneur.2025.1504535 (PMC11876041; doi:10.3389/fneur.2025.1504535)
Supplement: Supplementary file 1 [file Data_Sheet_1.pdf]

Supplementary Material

1 IMPLEMENTATION DETAILS

Further details can be found in the GitHub repository at `https://github.com/Wieske/DSA_comparison`.

1.1 MFPCA

We used the MFPCA implementation from the Python package FDApy [1], with some adaptations to enable the prediction of scores on new data with the PACE method.

1.2 Neural networks

We used Pytorch Paszke et al. (2019) for the implementation of the neural networks. We created a two-layer RNN with an output size equal to 8 for the synthetic datasets and 20 for ADNI. For the longitudinal prediction we created 2 linear layers with ReLU activation function and 30% probability of dropout. For the survival prediction we created 2 linear layers with 32 hidden nodes and 30% probability of dropout. For the loss function we used a learning rate of 1e-3 and included weight decay with a value of 1e-5. We trained the models with a batch size of 32 for 100 epochs.

1.3 Survival models

We used scikit-survival Pölsterl (2020) for the Cox Proportional Hazards model and the Random Survival Forest. For the Cox Proportional Hazards model we used the CoxPHSurvivalAnalysis model with tied events handled using the Efron method. For the Random Survival Forest we used the RandomSurvivalForest model with 1000 estimators and setting the minimum number of samples in a leaf node at 16. Other parameters were set at their default value.

[1] `https://fdapy.readthedocs.io/en/latest/`

2 DERIVATION OF SIMULATION SCENARIO 1

Here we will show that, given the longitudinal trajectories of scenario 1, it is possible to rewrite the time component of the covariates into the baseline hazard.

In scenario 1 the submodel for the longitudinal covariates is given by:

$$X_{iq}(t_{ij}) = \beta_{0q} + \beta_{1q}x_{iq} + \beta_{2q}t_{ij} + b_{iq},$$

and the hazard function is given by

$$h_i(t) = h_0(t)\exp[\gamma Z_i + \sum_{q=1}^{3}\alpha_q X_{iq}(t_{ij})].$$

If we fill the longitudinal submodels in this equation for the hazard we get:

$$\begin{aligned}
h_i(t) &= h_0(t)\exp[\gamma Z_i + \sum_{q=1}^{3}\alpha_q(\beta_{0q} + \beta_{1q}x_{iq} + \beta_{tq}t_{ij} + b_{iq})] \\
&= h_0(t)\exp[\gamma Z_i + \sum_{q=1}^{3}\alpha_q(\beta_{0q} + \beta_{1q}x_{iq} + b_{iq}) + \sum_{q=1}^{3}\alpha_q(\beta_{tq}t_{ij})] \\
&= h_0(t)\exp[\gamma Z_i + \sum_{q=1}^{3}\alpha_q(\beta_{0q} + \beta_{1q}x_{iq} + b_{iq}) + (\alpha_1\beta_{01} + \alpha_2\beta_{02} + \alpha_3\beta_{03})t_{ij}] \\
&= h_0(t)\exp((\alpha_1\beta_{01} + \alpha_2\beta_{02} + \alpha_3\beta_{03})t_{ij})\exp[\gamma Z_i + \sum_{q=1}^{3}\alpha_q(\beta_{0q} + \beta_{1q}x_{iq} + b_{iq})] \\
&= \exp(-7 + 0.74t_{ij})\exp[\gamma Z_i + \sum_{q=1}^{3}\alpha_q(\beta_{0q} + \beta_{1q}x_{iq} + b_{iq})]
\end{aligned}$$

In this scenario the baseline hazard $h_0(t) = \exp(-7)$ and $\alpha_1\beta_{01} + \alpha_2\beta_{02} + \alpha_3\beta_{03} = 0.74$. Therefore the description of scenario 1 is equal to a scenario based on only the values of the underlying covariates at baseline ($X_iq(t_{i0})$) with baseline hazard $h_0(t) = \exp(-7 + 0.74t_{ij})$.

REFERENCES

Paszke, A., Gross, S., Massa, F., Lerer, A., Bradbury, J., Chanan, G., et al. (2019). PyTorch: An Imperative Style, High-Performance Deep Learning Library. In *Advances in Neural Information Processing Systems 32*, eds. H. Wallach, H. Larochelle, A. Beygelzimer, F. d'Alché Buc, E. Fox, and R. Garnett (Curran Associates, Inc.), 8024–8035

Pölsterl, S. (2020). scikit-survival: A library for time-to-event analysis built on top of scikit-learn. *Journal of Machine Learning Research* 21, 1–6

3 SUPPLEMENTARY FIGURES

3.1 Simulation scenario 3

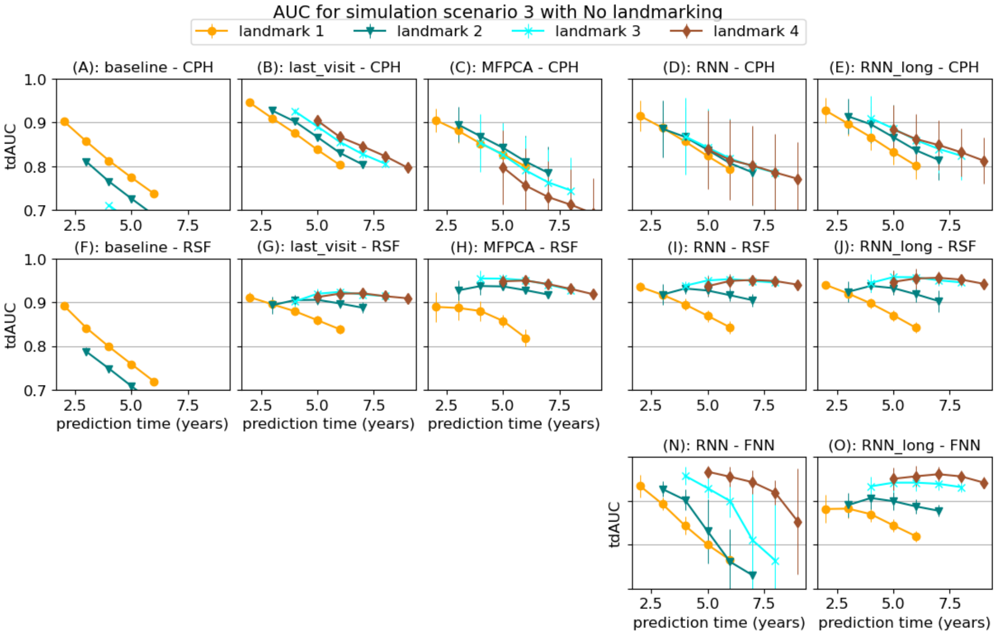

Figure S1. tdAUC across all model combinations for No landmarking method for simulation scenario 3.

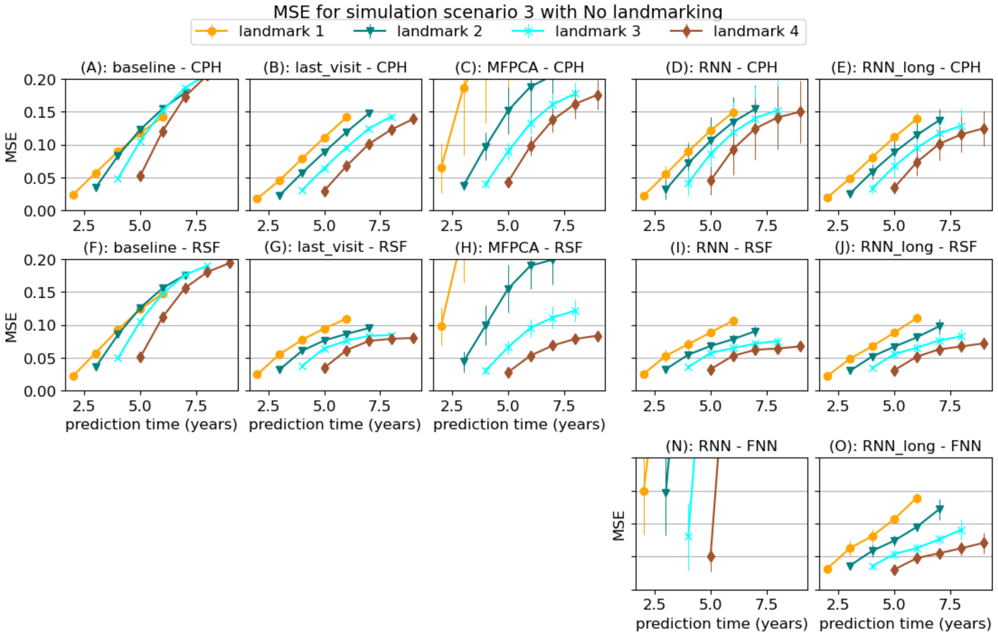

Figure S2. Mean Squared Error results across all model combinations for No landmarking method for simulation scenario 3.

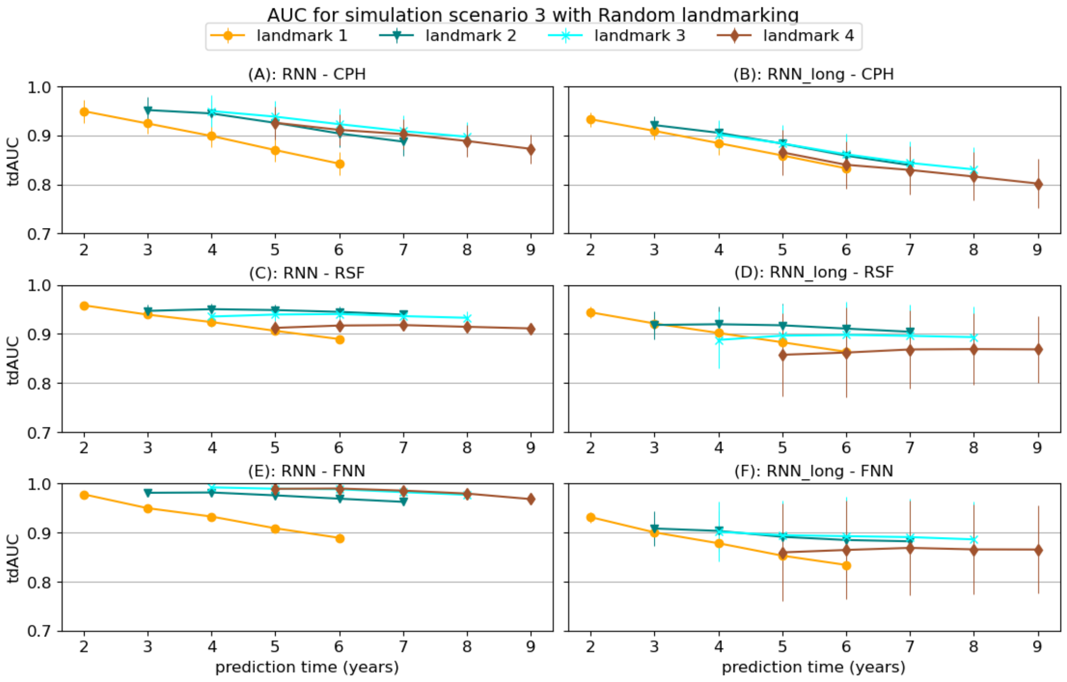

Figure S3. tdAUC across all model combinations for Random landmarking method for simulation scenario 3.

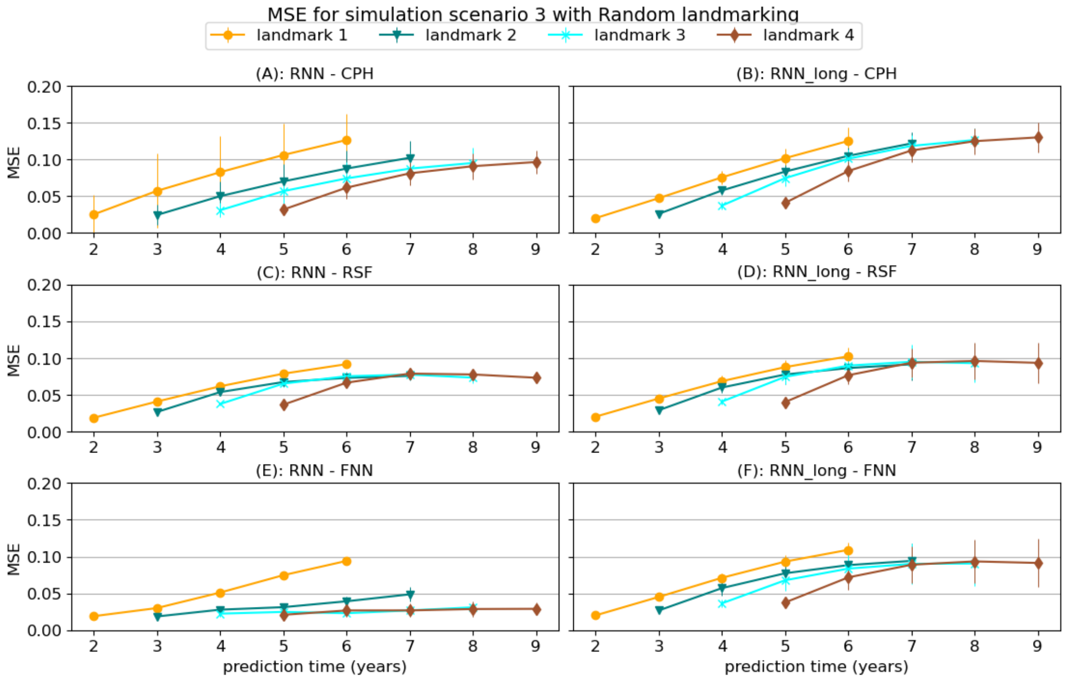

Figure S4. Mean Squared Error results across all model combinations for Random landmarking method for simulation scenario 3.

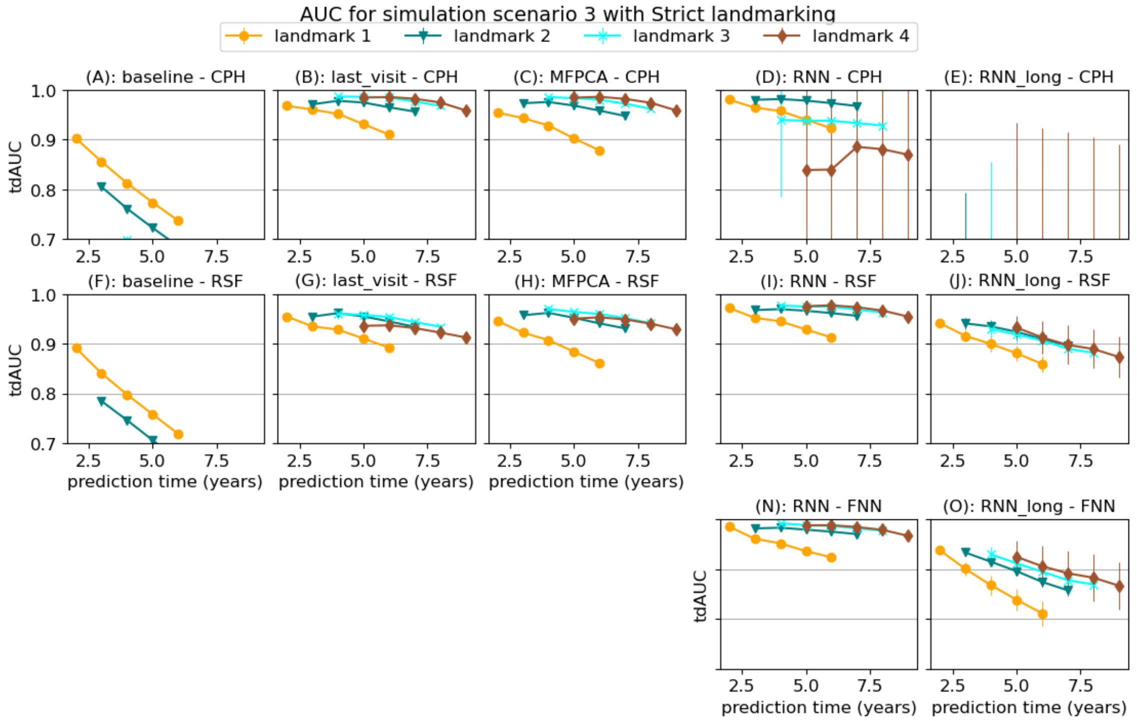

Figure S5. tdAUC across all model combinations for Strict landmarking method for simulation scenario 3.

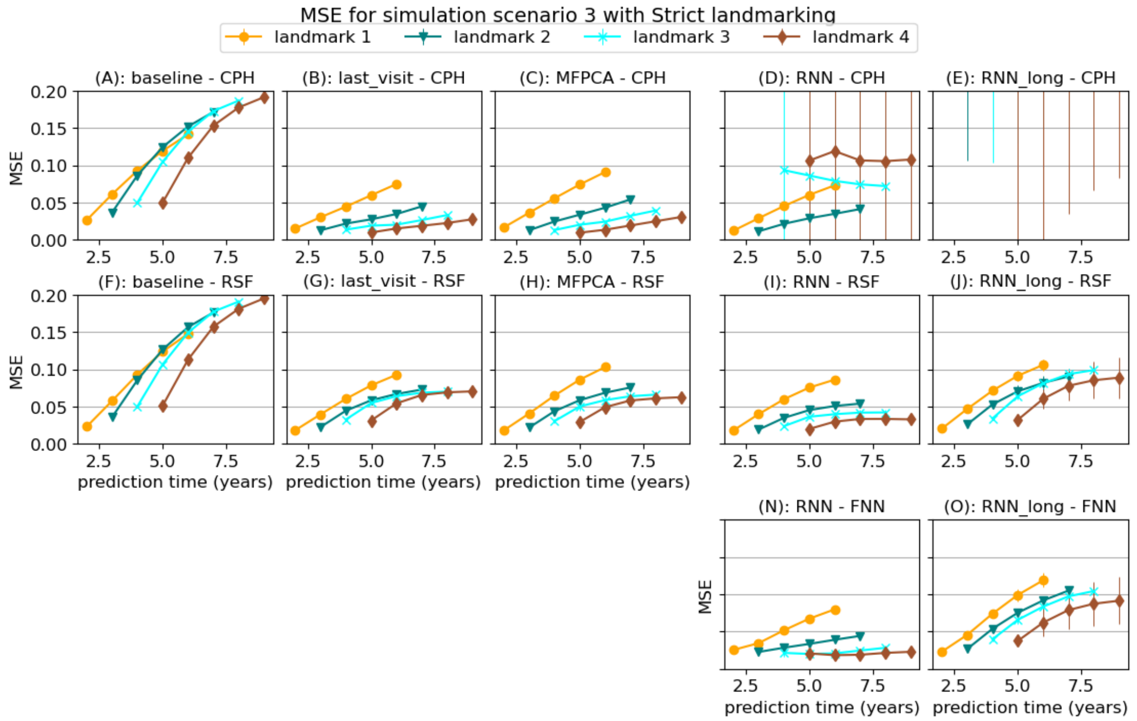

Figure S6. Mean Squared Error results across all model combinations for Strict landmarking method for simulation scenario 3.

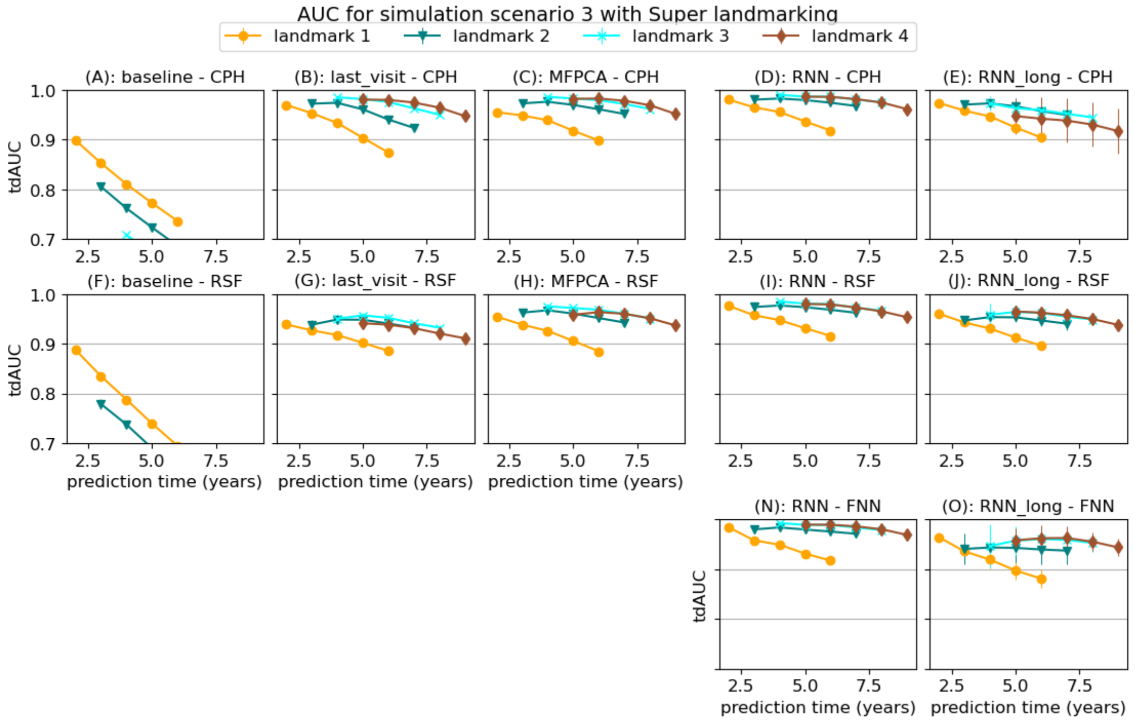

Figure S7. tdAUC across all model combinations for Super landmarking method for simulation scenario 3.

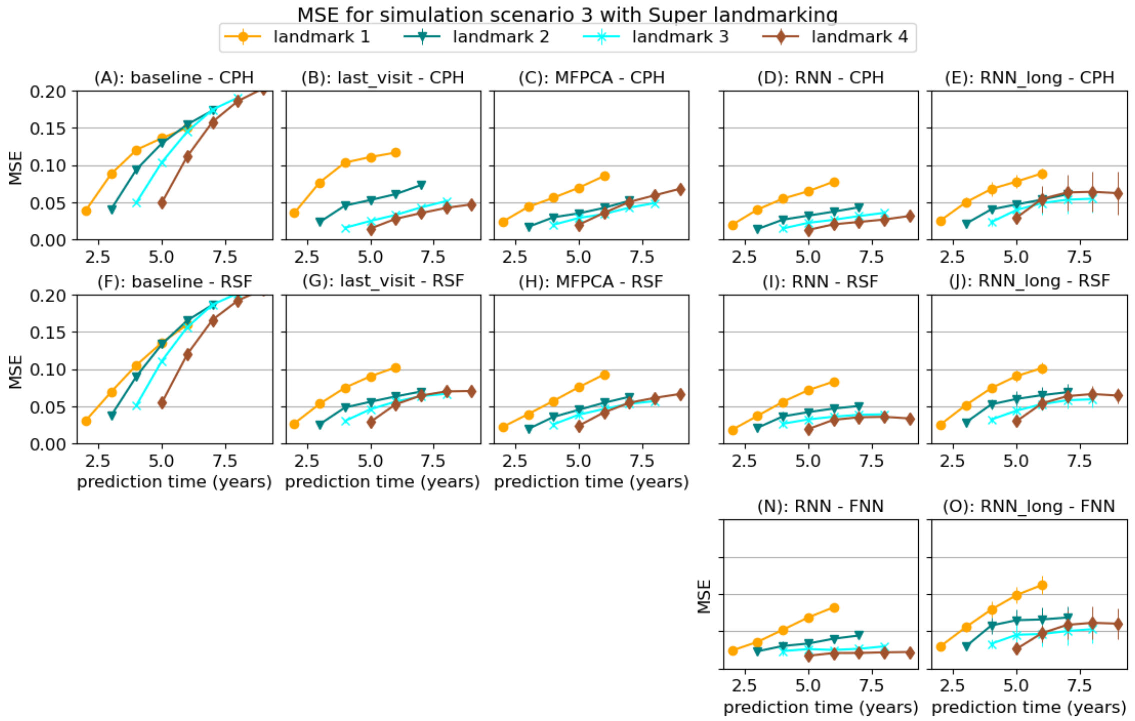

Figure S8. Mean Squared Error results across all model combinations for Super landmarking method for simulation scenario 3.

3.2 Application: ADNI

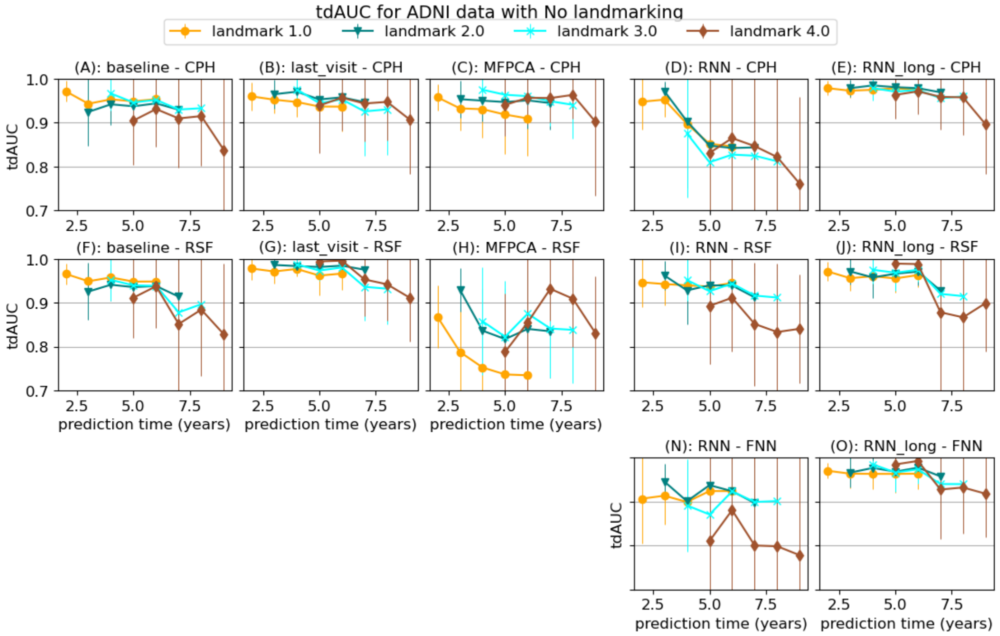

Figure S9. tdAUC across all model combinations for No landmarking method on the ADNI dataset.

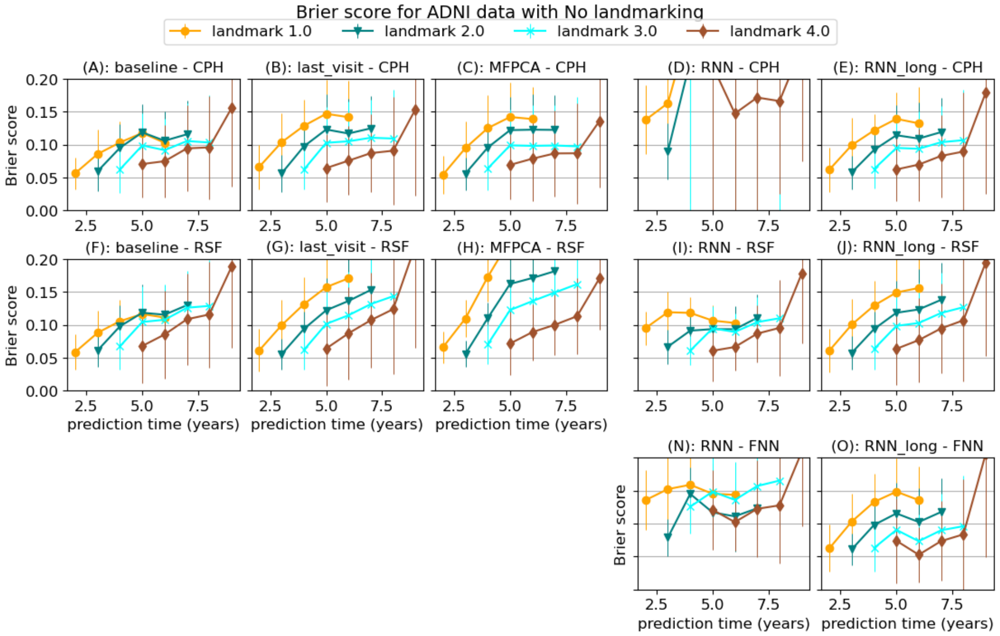

Figure S10. Brier score results across all model combinations for No landmarking method on the ADNI dataset.

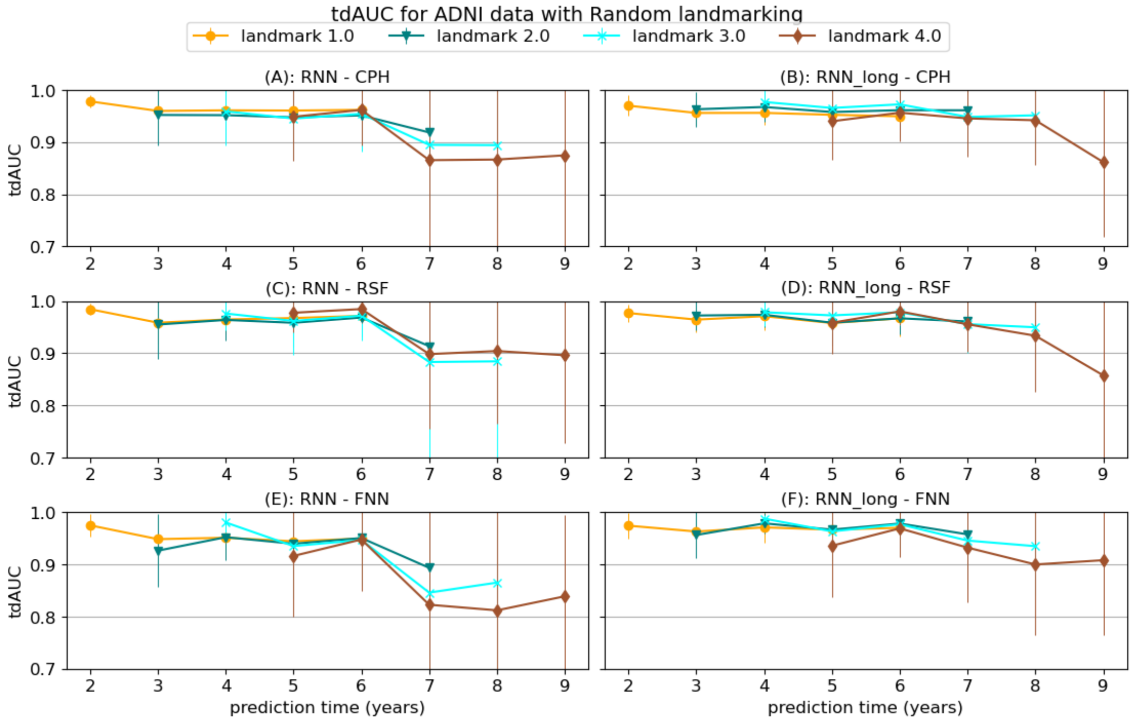

Figure S11. tdAUC across all model combinations for Random landmarking method on the ADNI dataset.

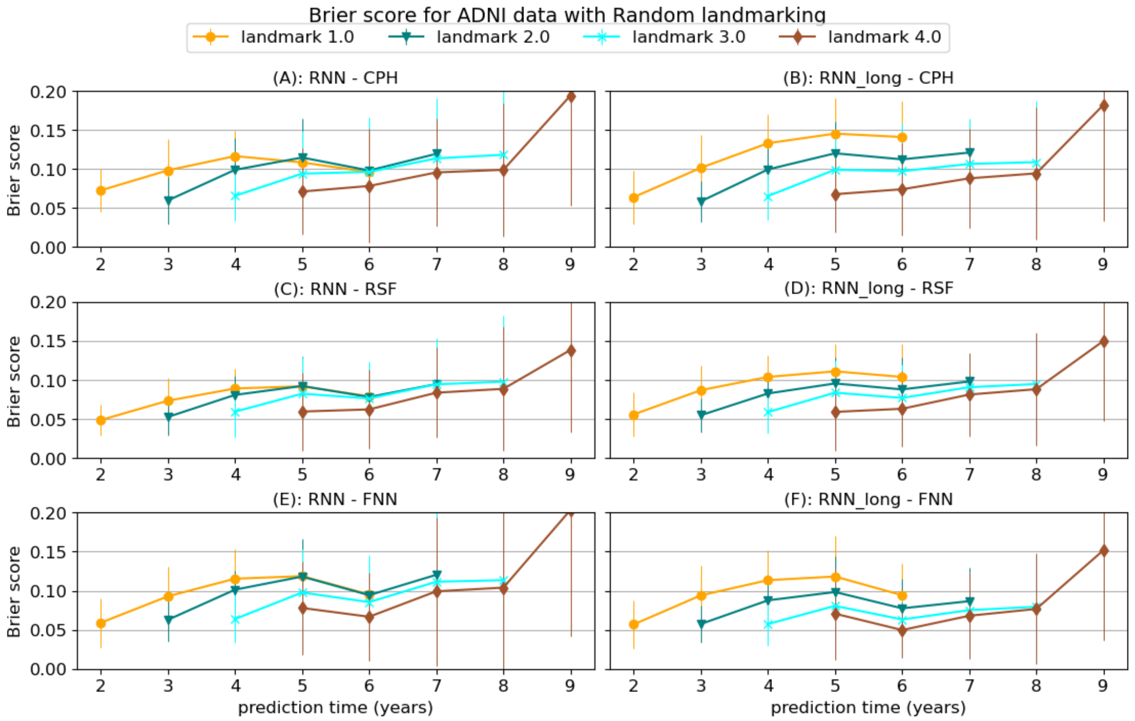

Figure S12. Brier score results across all model combinations for Random landmarking method on the ADNI dataset.

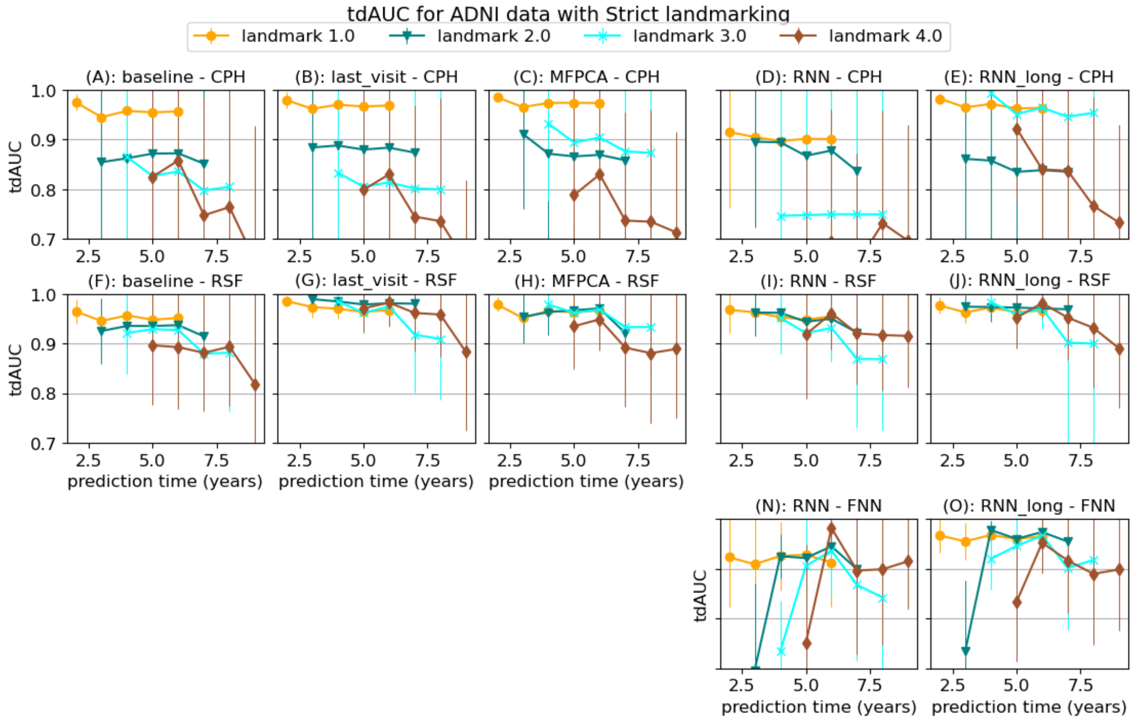

Figure S13. tdAUC across all model combinations for Strict landmarking method on the ADNI dataset.

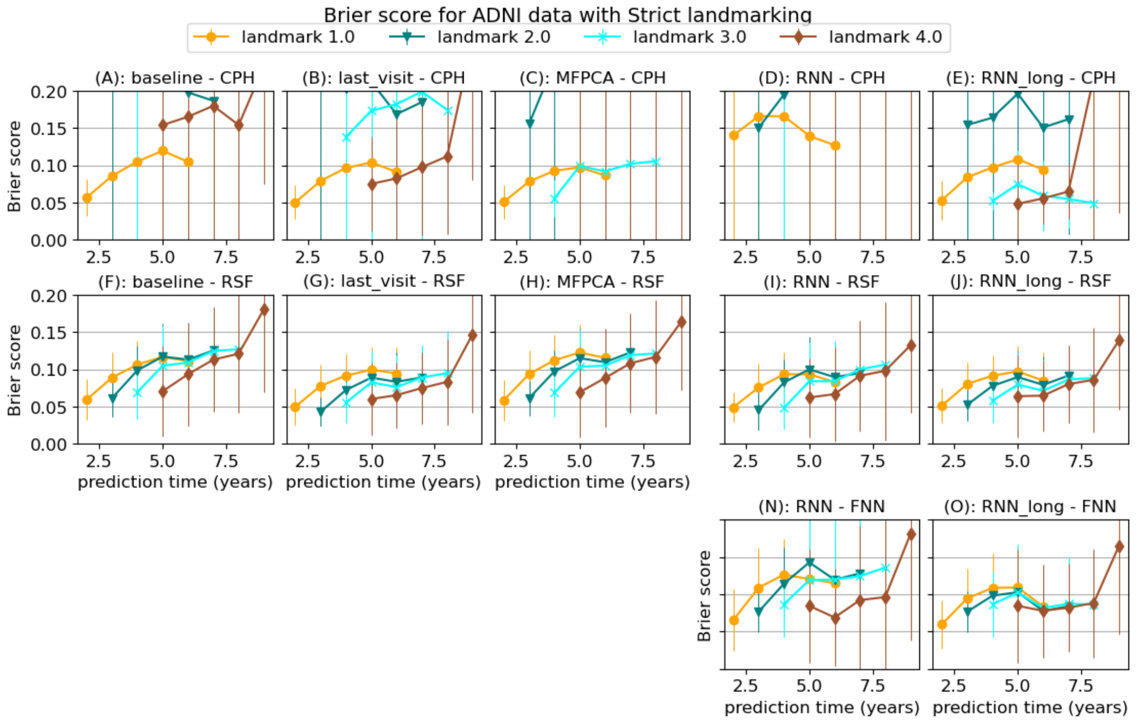

Figure S14. Brier score results across all model combinations for Strict landmarking method on the ADNI dataset.

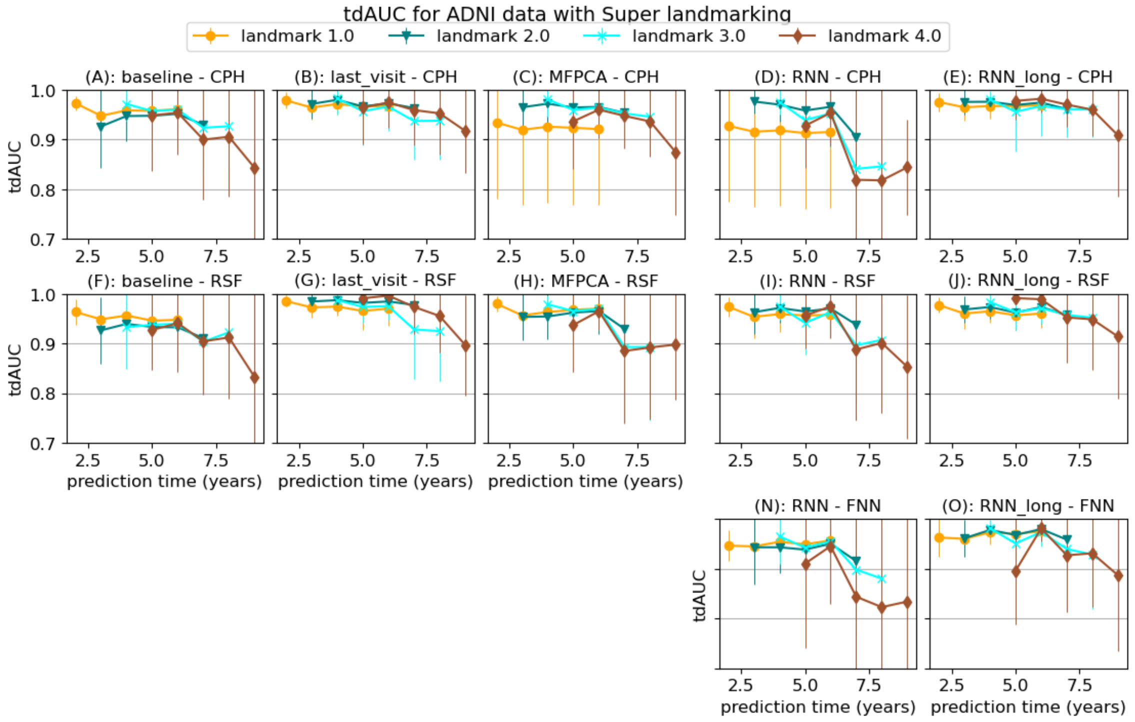

Figure S15. tdAUC across all model combinations for Super landmarking method on the ADNI dataset.

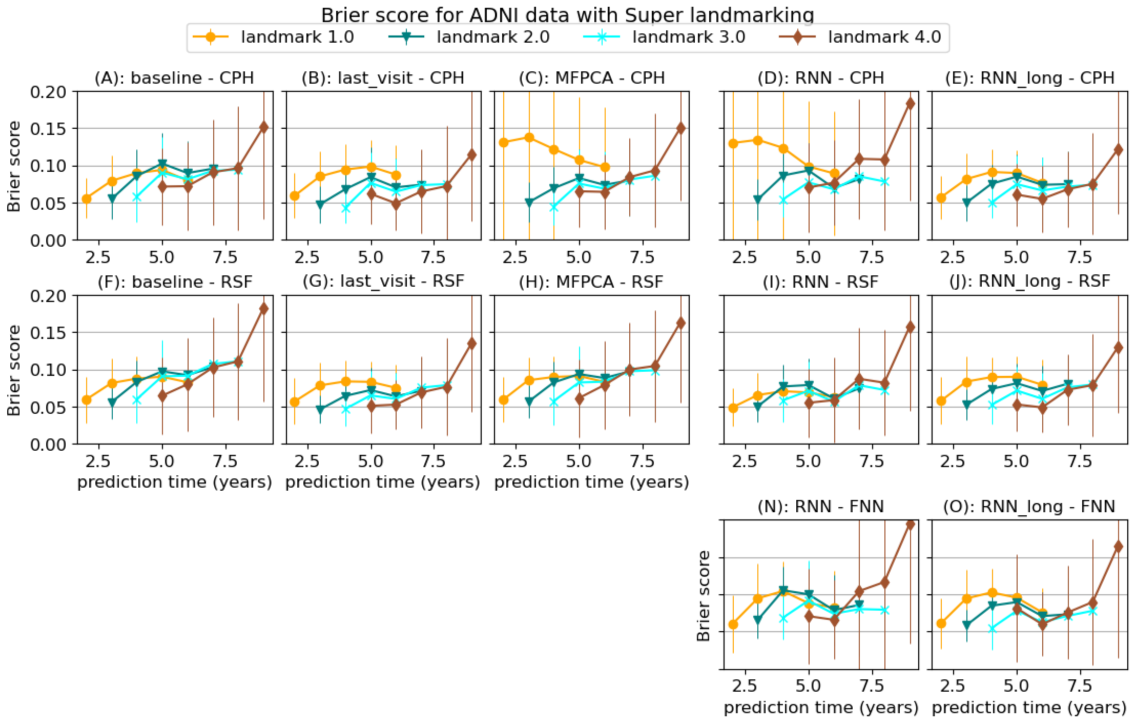

Figure S16. Brier score results across all model combinations for Super landmarking method on the ADNI dataset.
